# The Biomarker Toolkit — an evidence-based guideline to predict cancer biomarker success and guide development

**DOI:** 10.1186/s12916-023-03075-3

**Published:** 2023-10-04

**Authors:** Katerina-Vanessa Savva, Michal Kawka, Bhamini Vadhwana, Rahul Penumaka, Imogen Patton, Komal Khan, Claire Perrott, Saranya Das, Maxime Giot, Stella Mavroveli, George B. Hanna, Melody Zhifang Ni, Christopher J. Peters

**Affiliations:** Division of Surgery, Department of Surgery and Cancer, Imperial College London, London, UK

**Keywords:** Breast cancer, Biomarkers, Translational research, Clinical utility, Colorectal cancer

## Abstract

**Background:**

An increased number of resources are allocated on cancer biomarker discovery, but very few of these biomarkers are clinically adopted. To bridge the gap between Biomarker discovery and clinical use, we aim to generate the Biomarker Toolkit, a tool designed to identify clinically promising biomarkers and promote successful biomarker translation.

**Methods:**

All features associated with a clinically useful biomarker were identified using mixed-methodology, including systematic literature search, semi-structured interviews, and an online two-stage Delphi-Survey. Validation of the checklist was achieved by independent systematic literature searches using keywords/subheadings related to clinically and non-clinically utilised breast and colorectal cancer biomarkers. Composite aggregated scores were generated for each selected publication based on the presence/absence of an attribute listed in the Biomarker Toolkit checklist.

**Results:**

Systematic literature search identified 129 attributes associated with a clinically useful biomarker. These were grouped in four main categories including: rationale, clinical utility, analytical validity, and clinical validity. This checklist was subsequently developed using semi-structured interviews with biomarker experts (*n*=34); and 88.23% agreement was achieved regarding the identified attributes, via the Delphi survey (consensus level:75%, *n*=51). Quantitative validation was completed using clinically and non-clinically implemented breast and colorectal cancer biomarkers. Cox-regression analysis suggested that total score is a significant driver of biomarker success in both cancer types (BC: *p*>0.0001, 95.0% CI: 0.869–0.935, CRC: *p*>0.0001, 95.0% CI: 0.918–0.954).

**Conclusions:**

This novel study generated a validated checklist with literature-reported attributes linked with successful biomarker implementation. Ultimately, the application of this toolkit can be used to detect biomarkers with the highest clinical potential and shape how biomarker studies are designed/performed.

**Supplementary Information:**

The online version contains supplementary material available at 10.1186/s12916-023-03075-3.

## Background

There are a huge number of biomarker development studies, but most do not reach clinical practice [1–3]. In the context of cancer clinical research, a biomarker characterises the presence, progression or response to treatment. As biomarkers are implicated in all steps of patient care, there is an urgent need to reduce the translational gap between the bench side and clinic [3–5]. 

The exponential rise in the use of molecular profiling techniques, including metabolomics, genomics and proteomics, has not resulted in a corresponding rise in the number of clinically useful biomarkers. This emphasises that patients are yet to benefit from these discoveries. It is therefore vital to understand: (i) why biomarkers stall and (ii) how research focus needs to shift to promote successful utilisation of biomarkers.

Currently, there is no standard method to assess biomarker research trajectory and guide development; this is reflected by the excessive number of stalled biomarkers. There is a plethora of guidelines used to evaluate the quality/guide research design and reporting; however, in most cases, they provide generalised statements for specific study types. For example, STARD contains a list of essential items for reporting diagnostic accuracy studies [6, 7]. Similar to REMARK, STARD is vague in some instances by asking authors to provide enough detail to ‘allow replication’, without specifying what this entails. Replication is an extremely important characteristic enabling the researcher to achieve reproducible results, and meaningful outcomes; however, the lack of specific detail and explanation regarding this point might increase the risk of assessor bias/misjudgement when applying the checklist.

There is a clear need for detailed guidance to promote biomarker progression across the pipeline and avoid unnecessary and excessive research. Hypothesising that biomarkers unlikely to be clinically translated, lack certain attributes/characteristics in comparison to successfully implemented biomarkers, we aim to develop the Biomarker Toolkit. The Toolkit will consist of a checklist with literature-reported attributes/characteristics associated with a successful biomarker. For the purpose of this paper, a successful biomarker is defined as a biomarker that has been approved by national/international guidelines and is clinically used. On the other hand, a stalled biomarker is defined as a biomarker which is currently not clinically utilised/is not recommended for clinical use by national/international guidelines.

We initially identified the attributes of a successful biomarker through a literature search, semi-structured interviews, and a two-stage online Delphi survey with stakeholders. We subsequently validated the toolkit using published literature on breast and colorectal cancer biomarkers.

Upon validation, utilisation of the Biomarker Toolkit will ultimately define the characteristics of successful cancer biomarkers and will therefore begin to shape how biomarker studies are performed. We envision that the development of this novel tool will assist guidance of research trajectory from any stage of development to promote their clinical utilisation.

## Methods

The Biomarker Toolkit creation consists of two main components (i) development and (ii) validation. Firstly, we describe the development of the Biomarker Toolkit which is achieved by systematic literature search, semi-structured interviews and a two-round Delphi survey. Then we describe the methodology for validating the Biomarker Toolkit, utilising breast and colorectal biomarker literature.

### Development of the Biomarker Toolkit

#### Systematic literature search

An electronic systematic literature search was designed based on Preferred Reporting Items for Systematic Reviews and Meta-Analyses (PRISMA) guidelines [8]. The search was conducted in Ovid, using Medline and Embase databases. Keywords/subheadings used to identify relevant literature from 1946 to 2019 are described in Additional file: Table S1. Exclusion criteria included conference abstracts, studies not written in the English language, molecular biology primary studies, and literature addressing technical aspects of biomarkers. Inclusion criteria included reviews, expert commentaries, and surveys describing clinically useful cancer biomarkers. A detailed search was also conducted in the equator network (http://www.equator-network.org/), a website with the most frequently used reporting guidelines for health research (Additional file: Table S2). Identified articles and guidelines were screened by two reviewers (KVS and RP) to identify and extract attributes associated with successful biomarker implementation.

#### Semi-structured interviews and two-stage Delphi survey

Semi-structured interviews were conducted to (i) get the opinion of biomarker experts regarding the identified attributes from the systematic literature search and (ii) identify additional attributes to be added in the Biomarker Toolkit. The list of attributes was simplified using Table 1 as a reference and was presented to 34 biomarker experts/patient representatives. Demographic details are shown in Additional file: Table S3. A two-stage online Delphi survey was completed via Qualtrics where 54 biomarker experts participated. This survey aimed to achieve consensus on attributes related to successful biomarker implementation (75% agreement level — participant demographics shown in Additional file: Table S4). Detailed methodology of semi-structured interviews and Delphi survey was previously described by Huddy et al. (2018) and can also be found in Additional file: methods [9–17].

**Table 1:** Sub-category of attributes associated with a clinically useful biomarker

Analytical Validity	Clinical Utility
1) Analytical Modelling 2) Specimen Anatomical or Collection Site 3) Assay Validation/Precision/ Reproducibility/Accuracy 4) Quality Assurance of Reagents 5) Study specifies the assay method used 6) Study states whether Assay is Validated/Standardised/Optimised 7) Bio-specimen Matrix/Type 8) Biospecimen Collection 9) Biospecimen Inclusion/Exclusion Criteria 10) Biospecimen Quality 11) Cell Culture 12) Experimental Animal Details 13) Experimental Procedure Description 14) Mechanism of Stabilization 15) Sample Pre-processing 16) Storage/Shipping Transport 17) Time between Diagnosis and Sampling	34) Authority/Guideline Approval 35) Decisional Analysis 36) Cost-effectiveness 37) Ethics 38) Feasibility/BM Implementation 39) Funding 40) Harms and Toxicology 41) Invasiveness 42) Human Factor 43) Biomarker Usefulness 44) Study Type
Clinical Validity	Rationale
18) Adverse Events 19) Vital state of Biospecimen 20) Analytical Modelling 21) Blinding 22) Experimental Outcomes 23) Intervention 24) Missing Data 25) Methodology Details 26) Patient Eligibility 27) Reported Pre-specified Hypothesis 28) Randomisation 29) Reference Standard 30) Sample size Calculation 31) Sensitivity/ Specificity 32) Statistical Modelling 33) Trial Design Description	45) Identify the unmet clinical need for specific disease 46) Verify biomarker unmet need - is there an existing solution? 47) Pre-specified hypothesis/Exploratory discovery 48) Unmet need for the specified BM type

#### Score generation

As previously described, we aimed to identify characteristics related to a clinically useful biomarker. These characteristics form the basis of the Biomarker Toolkit, and their reporting in biomarker-related publications is used to mediate score calculation. Specifically, we firstly conducted a systematic literature search to identify relevant clinical articles, for the biomarker of interest. Subsequently, each selected clinical publication is read and scored based on the presence of the specified characteristics. The scoring system is binary. For example, if an attribute from the list is reported in a publication, that attribute is positively scored “1”. Conversely, if the attribute is not present, “0” is assigned. Average analytical validity, clinical validity and non-amended clinical utility scores are then calculated based on the average score of attributes listed under each individual category. The score provides a quantitative metric which reflects the accuracy of reporting, under each category addressed. Clinical utility score is then amended by taking into consideration the presence of additional study types, in addition to clinical studies (i.e., cost-effectiveness, human factor and implementation studies for the biomarker of interest), according to the formula detailed in Additional file: methods. In summary, scores for each biomarker being assessed are calculated using a four-step process which can be found in Additional file: methods.

### Validation of the Biomarker Toolkit

A successful biomarker is defined as a biomarker that has been approved by national/international guidelines and is clinically used. Breast and colorectal cancer biomarker fields were selected as validation platforms, because of the high level of research and the presence of at least some clinically implemented biomarkers. Identification and selection of successful and stalled breast and colorectal cancer biomarkers were completed from clinical guidelines [7–9, 18, 19]. Medline and Embase were used to collect primary literature from 1946 to 2019 using relevant keywords/subheadings (Additional file: Table S5 and S6). Biomarkers selected were approved by two oncological specialists in breast and gastrointestinal cancer. Breast and colorectal cancer biomarker search inclusion criteria include the following: (i) clinical studies where tumour specimens were prospectively/retrospectively collected, (ii) utility, (iii) decisional impact, (iv) cost-effectiveness, (v) feasibility/implementation studies, (vi) assay validation and (vii) human factor studies associated with the biomarker test. Exclusion criteria included studies not published in English language, conference abstracts, reviews, editorials, case studies, commentaries, and letters. Mendeley, referencing software was utilised to remove duplicate citations in all systematic searches. All systematic literature search results were screened by two reviewers (breast cancer: KVS and BV and CRC: KVS and MK).

### Statistical analysis

D’Agostino normality test was initially utilised to assess the normality of the data. Mann-Whitney *U* test was used to detect differences between successfully implemented and stalled biomarkers in clinical validity, analytical validity and clinical utility categories. Cox-regression analysis and binary logistic regression were performed to assess the effect of biomarker implementation status with scores from (i) sub-categories, (ii) main category of attributes (analytical validity, clinical validity, clinical utility) and (iii) total % scores. Statistical analysis test justification can be found in Additional file: methods [12, 20, 21]. Calculated *P*-value less than 0.05 was used as a reference to denote significant differences amongst compared groups. IBM SPSS statistics 25 (Version 25.0. Armonk, NY: IBM Corp.) was utilised to complete logistic and Cox regression models while the remaining statistical analysis was conducted utilising GraphPad Prism 7 (La Jolla, CA, USA).

## Results

### Development of the Biomarker Toolkit

The literature search for successful biomarker attributes identified 5665 articles. Following the removal of duplicate results and screening, 81 articles were selected (Additional file: Fig. S1). Screening of all articles and guidelines, retrieved 129 attributes describing a successful cancer biomarker (Additional file: Table S7). These 129 attributes were grouped in 48 sub-categories, based on common themes. The 48 sub-categories were then merged into four main categories including analytical validity, clinical validity, clinical utility and rationale (Table 1, Additional file: Fig. S2a and S2b). We identified 51 attributes under the analytical validity category (39.54%), 49 under the clinical validity category (37.98%), 25 under clinical utility (19.38%) and 4 attributes were identified under rationale (3.10%). Table 1 shows the allocation of attribute sub-categories within each main category. Detailed attributes under each sub-category/category are found in Additional file: Table S7.

A total of 34 semi-structured interviews were conducted between Oct 2019 and Jan 2020. The recruitment rate from invite was 87.18% (Additional file: Table S3). The median interview length was 14 minutes (range from 5 min and 36 s to 28 min and 54 s). The analysis of participant transcripts resulted in thematic saturation (Additional file: Fig. S3). Semi-structured interviews identified three additional attributes including (i) use of machine learning to automate biomarker assessment, (ii) being able to apply the biomarker to a close member of the family and (iii) the ability of a biomarker to be reimbursed. These attributes were grouped under already existing sub-categories including (i) feasibility/implementation, (ii) human factors and (iii) cost-effectiveness. In the two-stage online Delphi survey, 84.31% of attributes reached 75% consensus. Eight attributes were moved into Delphi round 2, from which 6 were removed upon completion of the second round (Additional file: Table S8a and S8b). Starting with 129 attributes, we added 3 according to semi-structured interviews and removed 6 upon Delphi survey completion. This resulted in 126 attributes falling into 47 sub-categories.

### Validation of Biomarker Toolkit

#### In breast cancer

A total of 377 publications were identified for successful biomarkers by abstract screening (MammaPrint:71, OncoTypeDX:251, PAM50:35 and Endopredict:20). Appropriate stalled biomarkers were selected to match the number of clinical studies identified in the successful group (Additional file: Table S5).

**Fig. 1:**
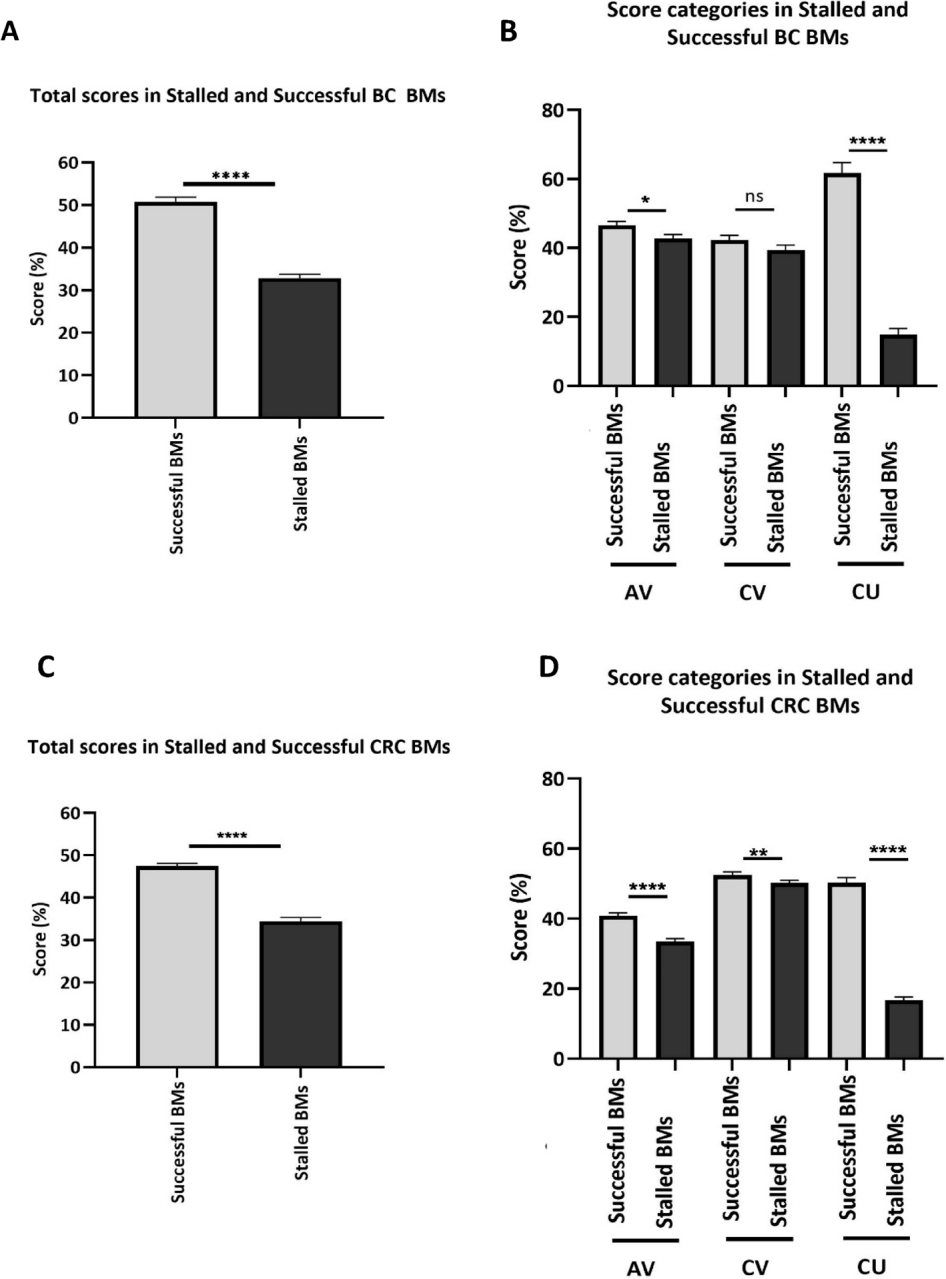
Scores and impact factor of successful and stalled breast and colorectal cancer BM publications. **A**) Bar chart indicating the total average scores between successful. (*n*=105) and stalled Biomarkers (*n*=80). **B**) Bar chart showing the individual average scores in CV, AV and CU categories. **C**) Bar chart indicating the total average scores between successful (*n*=132) and stalled CRC Biomarkers (*n*=123). **D**) Bar chart showing the individual average scores in CV, AV and CU categories. Asterisks denote the level of significance where ns: *P* > 0.05 *: *P*≤ 0.05, **: *P* ≤0.01, ***: *P* ≤0.001, ****: *P* ≤0.0001. All of the evaluated publications were independently scored by two assessors (BC: 50% of the journals by SM and 50% by MS; CRC 50% IP and 50% MK) as a validation of toolkit scoring strategy, with less than 12% difference between original and reviewer 1 and 2 scores, suggesting low inter-rater variability. AV, analytical validity; CV, clinical utility; CU, clinical utility; BM, biomarkers; CRC, colorectal cancer; BC, breast cancer

Rationale was not included in the validation because the selected biomarkers were of the same cancer type, therefore the rationale score would be similar for all evaluated biomarkers. Successful biomarkers have a significantly higher score compared to stalled biomarkers (Fig. 1A, D) using the Mann-Whitney *U* test (*P*>0.0001) and Cox regression model (*P*>0.0001; 95.0% CI for Exp(*B*): 0.869–0.935). Clinical utility score was the main driver of the difference seen in total scores, between the two groups (Fig. 1B, Cox regression *P*= 0.039 95.0% CI for Exp(*B*): 0.893–0.997). Clinical utility -related studies were conducted for only 6 out of the 32 stalled breast cancer biomarkers, and the frequency of publications was evidently lower in the stalled group (Additional file: Fig. S4 and S5). Regarding biomarker performance, only Negative Predictive Value was significantly lower in the stalled group (Fig. 3A).

Eight sub-categories were associated with biomarker success using binary logistic regression. These sub-categories include the following: (i) reporting of assay validation, (ii) use of a reference standard, (iii) study randomisation/blinding, (iv) detailed reporting of participant information, (v) reporting of methodological details, (vi) study adverse events and (vii) participant confounding factors in addition to (viii) biospecimen inclusion/exclusion criteria (Additional file: Table S9).

#### In colorectal cancer

CRC literature searches identified a total of 264 publications for successful biomarkers (KRAS:139, BRAF:125) and 138 for stalled biomarkers (PTEN:40, immunoscore:12, OncoDx:10, PD-L1:22 and PIK3CA:54).

Average total % scores of successful biomarkers were significantly higher in comparison to the stalled biomarker group, following the breast cancer pattern (Fig. 1/Mann-Whitney *U* test: *P*>0.0001/Cox regression analysis: *P*>0.0001, 95.0% CI for Exp(*B*): 0.918–0.954). All three main categories were significantly different between stalled and successful biomarkers, and like in breast cancer, the biggest driver of score difference was the clinical utility category (Cox regression analysis: *P*> 0.0001, 95.0% CI for Exp(*B*):0.949–0.969). There was an evident increase in frequency of decisional analysis, cost-effectiveness, implementation, feasibility, clinical usefulness, and human factor studies in successful biomarkers. In addition, lower clinical utility publication frequency was also seen when evaluating individual biomarkers (Additional file: Fig. S6).

Eleven sub-categories were significantly associated with biomarker success using binary logistic regression (Additional file: Table S10). These include, reporting of adverse event, assay validation aspects, biospecimen reporting, experimental procedures, patient eligibility and reference standard reporting within others. When assessing biomarker performance characteristics, none of end points were significantly different between the two groups (Fig. 3B).

## Discussion

This novel study utilised an extensive systematic literature search and currently established clinical guidelines to identify attributes defining a successful biomarker, creating the Biomarker Toolkit. Successful validation of the toolkit in both colorectal and breast cancer emphasised its potential as a useful tool for clinicians, academics and industry moving towards the identification of more clinically promising biomarkers.

The 126 attributes included in the Biomarker Toolkit were derived from a combination of systematic literature search results and health study-related guidelines. This greatly improved the clarity of the toolkit, by converging a wide range of attributes in a holistic way.

**Fig. 2:**
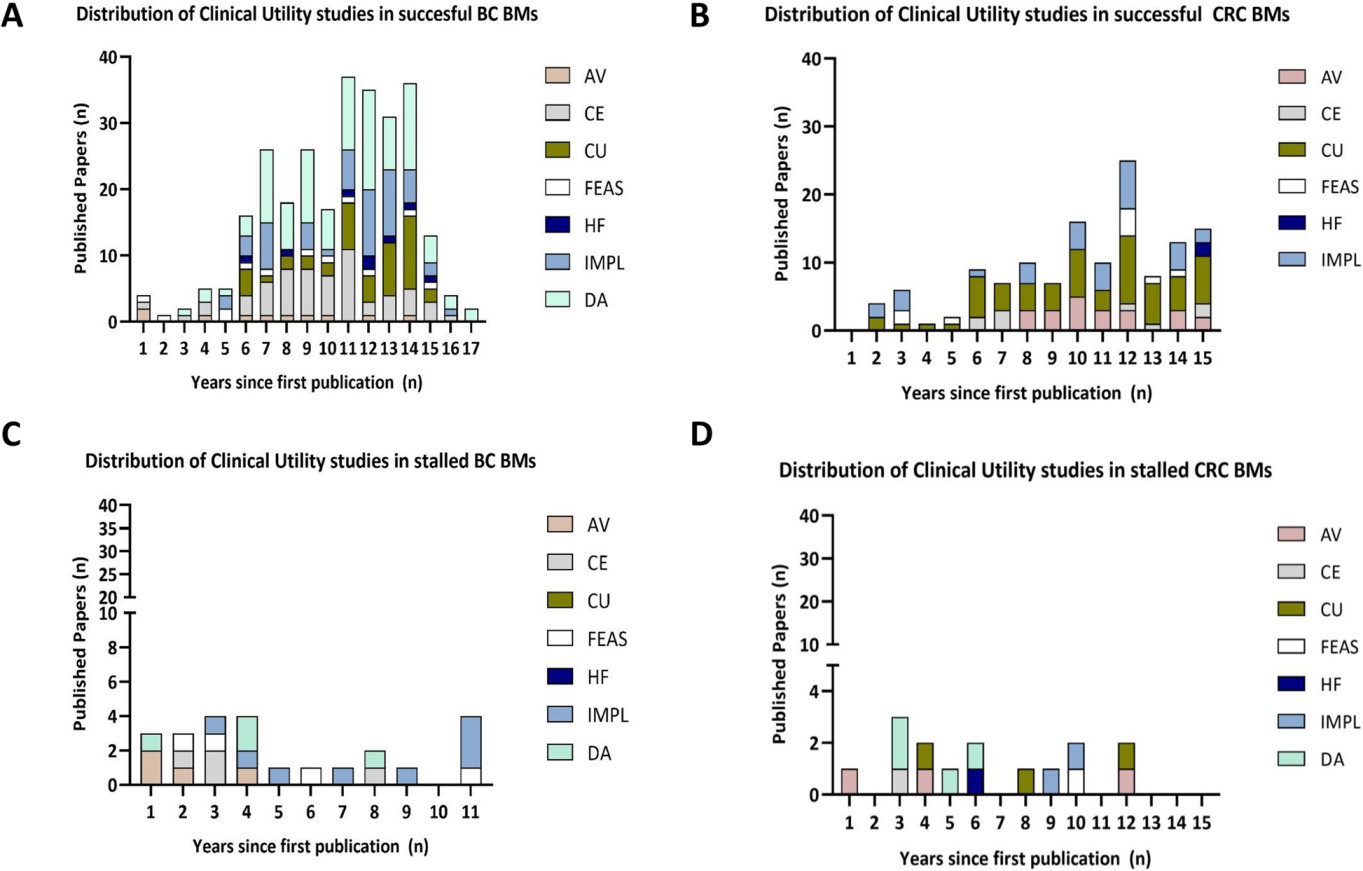
Successful and stalled biomarker clinical utility studies. Stacked bar chart showing AV, analytical validity; CE, cost-effectiveness; CUs, clinical usefulness; FEAS, feasibility; HF, human factor; IMPL, implementation; and DA, decisional analysis studies in successful **A**) BC, **B**) CRC biomarkers and stalled **C**) BC and **D**) CRC biomarkers. BC, breast cancer, CRC, colorectal cancer

To allow comparison between breast and colorectal cancer publication scores, the same set of attributes were assessed in both cases. No attributes were excluded to prevent risk of model overfitting. Significantly higher scores in the successful group of both breast and colorectal cancer biomarkers reinforce that the toolkit has the potential to distinguish successful from stalled biomarkers, in two independent cancer types (Figs. 1A and 2A).

As the clinical utility category appeared to be the main driving force behind successful biomarker scores, we aimed to study in detail the source of this difference. As indicated in Fig. 2, successful biomarkers appear to have an evidently higher number of clinical utility publications, published within shorter intervals. Publications appear to show a rise in number approximately 5 years upon biomarker discovery (Fig. 2 and Additional file: Fig. S4–6). This strongly highlights the need to build clinical utility study types, into research plans at this stage, if researchers aim to achieve clinical adoption. This also reinforces the critical need to guide researchers in utilising the Biomarker Toolkit to progress candidate biomarkers along the biomarker pipeline, rather than engaging with excessive biomarker discovery.

Specifically, the accuracy of reporting of assay validation metrics showed the greatest difference between successful and stalled biomarkers in both breast and colorectal cancer biomarker cohorts (Additional file: Tables S9 and S10). Experimental procedure description, method optimisation/validation and biospecimen inclusion/exclusion criteria were more frequently reported in successful biomarker publications in contrast to the stalled biomarkers, where they were more frequently omitted (Additional file: Tables S9 and S10). These attributes contributed to the highest impact towards the overall score. In agreement with the online Delphi survey stage 3, the analytical validity category was ranked as the most important across all biomarker types (Additional file: Table S11). This emphasises the importance of test standardisation and assay development which is often associated with assay commercialisation. Commercialisation of a specific test allows test standardisation and intra-inter-lab repeatability, emphasising the importance of these attributes at an early stage during biomarker development [22].

Currently, the Biomarker Toolkit assesses the totality of the literature for a specific biomarker and provides research guidance towards specified aspects of the field. For example, if the biomarker toolkit is evaluating an early-stage biomarker there will naturally be limited data to assess clinical utility in the early stages. Although a low score will be acquired in this category, it will encourage and guide the researcher to address specific clinical utility aspects and help improve their clinical potential and progression along the pipeline.

At this stage, scores were generated assuming equal weights for each attribute being addressed. This might generate a score with limited reliability as different categories might have a greater level of importance. We utilised the ranks from the online Delphi survey to add weights to different groups of attributes (data available upon request). Adding weights did not appear to cause significant changes in *p* values of the Cox regression models (Additional file: Tables S12 and S13). Thus, we decided to exclude the use of weightings in the calculations, as (i) they do not improve the Cox regression model in both cancer types while (ii) they add an unnecessary complexity to score calculation. Nevertheless, it is clear that different categories have varying levels of importance to each end user dictated by the remit of the biomarker. Future work on the Biomarker Toolkit aims to enable the user to select weightings for each category of attributes to tailor scoring to meet the goals of the end user. At this stage, we validated the Biomarker Toolkit checklist using analytical validity, clinical validity and clinical utility. Rationale was excluded from study validation as we compared biomarkers of the same cancer type. Future versions of the toolkit aim to include quantitative comparative analysis based on biomarker rationale-related attributes, to assist in the prioritisation of biomarkers with a greater unmet need. Most aspects addressed under the rationale category are subjective and will depend on practice, experience, interest of stakeholders, intended country of use, cancer type, biomarker type in other factors. Incorporating these findings into the Biomarker Toolkit scores will enable the user to evaluate biomarker potential, according to the specific needs of the user [23].

Future work also aims to validate the toolkit using industrial collaborator biomarker databases. By assessing additional biomarkers, we can define cut-off values between successful and stalled biomarkers. This threshold will be used as a reference to compare scores of newly/prospectively assessed biomarkers.

In addition to KRAS and BRAF, microsatellite instability (MSI) has also been demonstrated to be a clinically valuable biomarker [24]. A systematic literature search for KRAS and BRAF yielded a total of 132 clinical papers. Future work aims to address additional biomarkers to enrich the score databank and enable model training utilising natural language processing.

This study generated the first version of the toolkit which requires human input; however, the ultimate vision is to automate the Biomarker Toolkit and establish an accessible application. A specific biomarker will be defined by the user, and all relevant publications will be automatically identified and retrieved from databases. All publications will be automatically screened and scored based on the presence of Biomarker Toolkit attributes. In this way, derived scores can be compared between different biomarkers to assess their clinical potential.

There are various barriers preventing biomarkers from being clinically useful, as previously discussed. Biomarker toolkit scores in individual subcategories reflect gaps in research for a specific biomarker and therefore guide research and development, even at an early stage. This will undoubtedly improve the research quality of reporting and promote the clinical utilisation of biomarkers.

The Biomarker Toolkit is designed to evaluate biomarkers which show early promise, are stalled or slowed down or progressing towards later stages of the pipeline. Future work aims to further develop the Toolkit to allow an individual prediction of the likelihood of a biomarker’s success at each stage of the biomarker’s development based on the scores of each individual category.

**Fig. 3:**
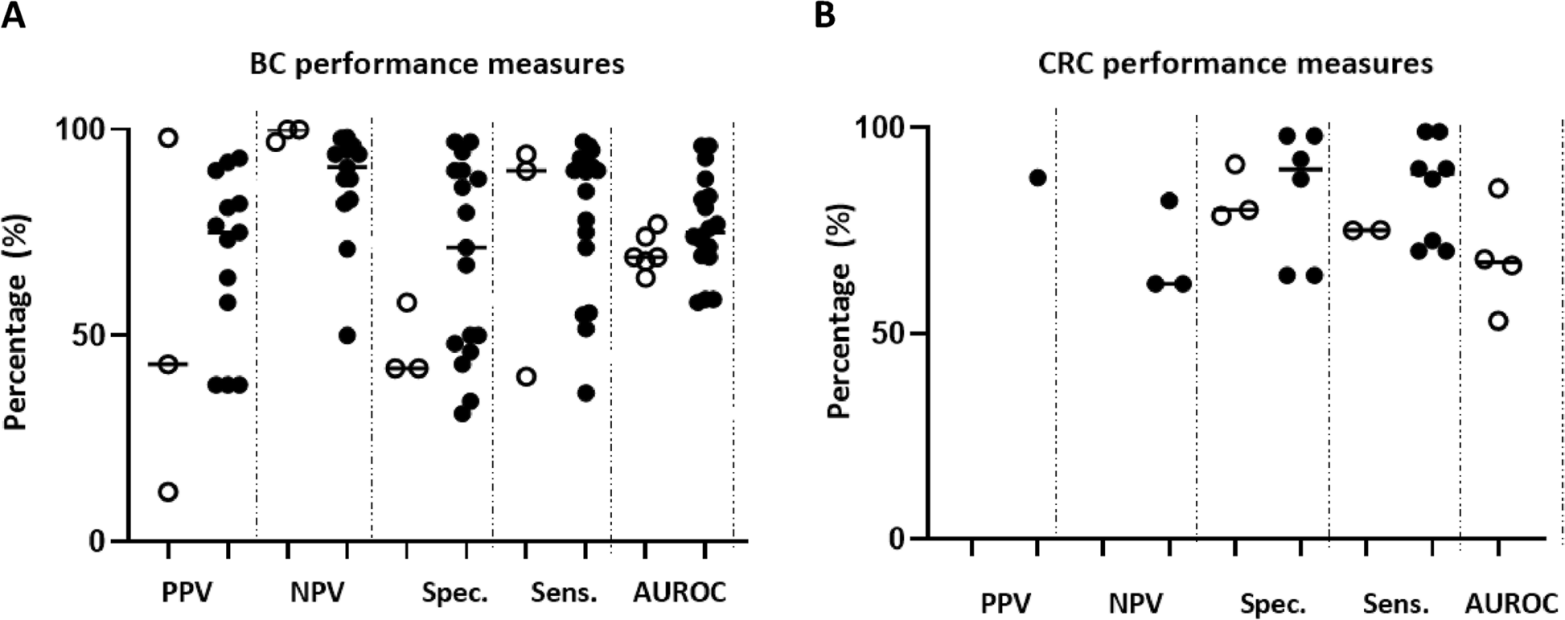
Performance outcomes in successful and stalled breast and CRC biomarkers studies: scatter plot showing PPV, positive predictive value; NPV, negative predictive value; Spec., specificity; Sens, sensitivity; AUROC, area under the curve in **A**) BC and **B**) CRC successful (open circles) and stalled (closed circles) biomarkers. BC, breast cancer, CRC, colorectal cancer

This study focused on published literature which is a potential limitation. Specifically, as negative findings are not published, they are therefore impossible to assess. It should be noted that scores are based on literature-reported attributes which do not necessarily reflect biomarker performance. Evidently, a high sensitivity and specificity threshold is of vital importance for a successful biomarker however, there were no significant differences in the performance characteristics assessed between successful and stalled biomarkers, suggesting that these measures do not provide a useful discriminator for biomarker success on their own (Fig. 3). In most cases, only positive results are reported in literature irrespective of a biomarker’s clinical potential. This emphasises the importance of the toolkit, which enables the user to assess a wide range of aspects from biomarker publications, in addition to the commonly addressed performance attributes.

Currently, there is no standardised tool to enable the assessment of biomarkers and target research to those more likely to be clinically translated. There are enormous time and cost implications due to excessive research with very little clinical impact. As supported by Savva et al., a systematic literature search identified 77 gastrointestinal cancer biomarker panels, including 25 with an AUROC >0.9. From the identified biomarkers, all except one were stalled at the discovery phase and none were clinically utilised. This supports the presence of a translational gap, despite high clinical biomarker performance [25].

## Conclusions

This original paper focuses on the development and validation of the Biomarker Toolkit. This is a novel tool which encourages a multi-dimensional approach to assess cancer biomarker clinical potential, thus improving the fragmented pathway towards biomarker clinical translation.

The Biomarker Toolkit has the potential to act as a framework that funders, researchers, academics, clinicians and industry can all use in a consistent manner to (i) improve research quality, (ii) standardise biomarker research while (iii) assessing which biomarkers have higher potential and should therefore get funded. It also has the power to rescue stalled biomarkers by identifying where the evidence gaps lie and provide a roadmap for ongoing research. Ultimately, with this toolkit, industry, funding bodies and researchers will finally have a way to navigate biomarkers through the challenging journey of clinical translation. This will reduce the cost and time associated with biomarker development.

### Supplementary Information

**Additional file 1:Table S1.** Search terms used in four systematic literature searches. **Table S2.** Guidelines used to extract characteristics associated with successful Biomarkers. **Table S3.** Semi-structured interview participant demographics. **Table S4.** Delphi Participant Demographics. **Table S5 & S6.** Table indicating a modified version of PRISMA flow diagram for breast and colorectal cancer. **Table S7.** Detailed Attributes extracted from systematic literature and guidelines. **Table S8a & S8b.** Result Summary of stage A- round 1 online Delphi Survey and Table indicating characteristics that moved into the second round of the Delphi Survey, and the % agreement reached in round 1 and 2, respectively. **Table S9 & S10.** Table showing the attribute-categories identified as significantly different between successful and stalled breast (S9) & CRC (S10) cancer biomarkers, using Man Whitney- U test and binary logistic regression*. **Table S11.** Table indicating the median rank for each subcategory (1-5), in Analytical Validity, Clinical Validity, Clinical Utility and Rationale for the seven indicated biomarker types (n=7). **Table S12 & S13.** Cox Regression Model for unweighted, weighted, and weighted top 3 categories, Breast Cancer (S12) & CRC (S13) Biomarker scores. **Figure S1**: PRISMA illustrating study selection for Biomarker criteria checklist. **Figure S2 & S2b.** S2a) Categorisation/Grouping of Biomarker toolkit Characteristics. S2b) Biomarker characteristics associated with successful biomarker clinical implementation. **Figure S3.** Themes identified via semi-structured interview thematic analysis. Thematic analysis and figures were constructed using Nvivo12 Pro.* Additional File Figure S4.* Successful Biomarker Clinical Utility Studies. **Figure S5 & S6.** Stalled Biomarker Clinical Utility Studies for BC (S5) and CRC (S6).

## Data Availability

All data presented in the study can be found in the main and the additional file.

## Abbreviations

AV: Analytical validity
BC: Breast cancer
BM: Biomarkers
CE: Cost-effectiveness
CRC: Colorectal cancer
CU: Clinical utility
CUs: Clinical usefulness
DA: Decisional analysis
FEAS: Feasibility
HF: Human factor
IMPL: Implementation
