# Supplementary material for: The Biomarker Toolkit — an evidence-based guideline to predict cancer biomarker success and guide development
Source: BMC Med. 2023 Oct 4;21:383. doi: 10.1186/s12916-023-03075-3 (PMC10552368; doi:10.1186/s12916-023-03075-3)
Supplement: Supplementary file 1 — Additional file 1:Table S1. Search terms used in four systematic literature searches. Table S2. Guidelines used to extract characteristics associated with successful Biomarkers. Table S3. Semi-structured interview participant demographics. Table S4. Delphi Participant Demographics. Table S5 & S6. Table indicating a modified version of PRISMA flow diagram for breast and colorectal cancer. Table S7. Detailed Attributes extracted from systematic literature and guidelines. Table S8a & S8b. Result Summary of stage A- round 1 online Delphi Survey and Table indicating characteristics that moved into the second round of the Delphi Survey, and the % agreement reached in round 1 and 2, respectively. Table S9 & S10. Table showing the attribute-categories identified as significantly different between successful and stalled breast (S9) & CRC (S10) cancer biomarkers, using Man Whitney- U test and binary logistic regression*. Table S11. Table indicating the median rank for each subcategory (1-5), in Analytical Validity, Clinical Validity, Clinical Utility and Rationale for the seven indicated biomarker types (n=7). Table S12 & S13. Cox Regression Model for unweighted, weighted, and weighted top 3 categories, Breast Cancer (S12) & CRC (S13) Biomarker scores. Figure S1: PRISMA illustrating study selection for Biomarker criteria checklist. Figure S2 & S2b. S2a) Categorisation/Grouping of Biomarker toolkit Characteristics. S2b) Biomarker characteristics associated with successful biomarker clinical implementation. Figure S3. Themes identified via semi-structured interview thematic analysis. Thematic analysis and figures were constructed using Nvivo12 Pro. Additional File Figure S4. Successful Biomarker Clinical Utility Studies. Figure S5 & S6. Stalled Biomarker Clinical Utility Studies for BC (S5) and CRC (S6). [file 12916_2023_3075_MOESM1_ESM.pdf]

Additional File Table S1: Search terms used in four systematic literature searches.			
	SEARCH TERMS	*MEDLINE*	*EMBASE*
**1**	exp Biomarkers, Tumor/	244390	
**2**	((cancer or tumo?r) adj3 biomarker*).mp. [mp=title, abstract, original title, name of substance word, subject heading word, floating sub-heading word, keyword heading word, organism supplementary concept word, protocol supplementary concept word, rare disease supplementary concept word, unique identifier, synonyms]	141127	24204
**3**	exp Translational Medical Research/	9465	
**4**	translation*.mp.	255328	279061
**5**	Clinical effectiveness.mp. or Treatment Outcome/	912234	918147
**6**	Clinical effectiveness.mp.	10384	122219
**7**	pipeline*.mp.	19014	27694
**8**	Clinical application*.mp.	77105	103379
**9**	(clinical* adj4 relevant).mp. [mp=title, abstract, original title, name of substance word, subject heading word, floating sub-heading word, keyword heading word, organism supplementary concept word, protocol supplementary concept word, rare disease supplementary concept word, unique identifier, synonyms]	73027	111573
**10**	utility.mp.	184411	259264
**11**	1 or 2	253003	
**12**	3 or 4	255328	
**13**	5 or 6 or 7 or 8 or 9 or 10	1241371	
**14**	11 and 12 and 13	336	
**15**	exp translational research/		15665
**16**	2 or 15		286660
**17**	4 or 16		279061
**18**	13 and 17 and 20		436

**19**	Oncotype DX or Oncotype-DX or Oncotype - DX or 12 gene or 21-gene or 21 - gene or recurrence score	2309	4791
**20**	MapQuant Dx or MapQuantDx or GGI or Genomic Grade Index or reduced Genomic Grade Index or reduced GGI or rGGI or GGI reduced or GGIr or 97-gen* or 97 gen*).	442	718
**21**	MammaPrint or Mamma-Print or Mamma Print or 70 gene signature or 70gene signature or 70-gene signature	209	674

Additional File Table S2: Guidelines used to extract characteristics associated with successful Biomarkers	
**Guideline Name**	**Study Type**
STARD	Diagnostic/prognostic studies
TRIPOD	Diagnostic/prognostic studies
REMARK	Tumour Marker Prognostic studies
ARRIVE	Animal pre-clinical studies
CHEERS	Economic evaluation
CONSORT	Randomised trials
STROBE	Observational studies
QUADAS2	Risk of bias and applicability of primary diagnostic accuracy studies

Additional File Table S3: Semi-structured interview participant demographics					
**Group**	**Academic**	**Clinician**	**Industry**	**CPR/S**	**Total**
Participant number	8	10	8	8	34
Sex (M: F)	(2:6)	(10:0)	(4:4)	(1:7)	(27:17)
Age (mean ± STDEV)	37.88 ± 6.38	44.5 ± 11.40	44.75± 11.62	64.75± 10.96	47.76 ± 14.05
*CPR/S: Cancer Patient Representatives/Survivors					

Additional File Table S4: Delphi Participant Demographics

**Years of experience (y ± STDEV)**	7.9 (± 7.69)		**Expertise n (%)**	
**Male: Female**	33:21		*Academia, industry*	1 (1.85)
**Age (±STDEV)**	42.66 (14.35)		*Academia*	15 (27.78)
**Ethnicity (%)**			*Academia, Clinician*	21 (38.89)
White	43 (79.63)		*Clinician*	7 (12.96)
Asian	6 (11.11)		*Industry*	4 (7.41)
Arab	2 (3.70)		*Research Institute*	4 (7.41)
Middle east	1 (1.85)		*Academia, Industry, Clinician*	2 (3.70)
Kurdish	1 (1.85)			
Other	1 (1.85)			

Additional File Table S5: Table indicating a modified version of PRISMA flow diagram. For simplicity and more effective representation of the large number of systematic searches, PRISMA flow diagram was tabulated. This tabulated PRISMA indicates details of systematic searches of 4 successful and 32 stalled breast cancer biomarkers.

	IDENTIFICATION				SCREENING	ELIGIBILITY	INCLUDED	
	All Articles	Embase	Medline	Extra articles	Records after duplicate removal	Full Articles assed for eligibility	Selected articles	Types of Articles selected*
264-gene signature or 264 gene signature or 264 gen* signature or 264-gen* signature or Novel2 or Novel 2	4,859	2,895	1,964	0	3,295	1	1	1C
26 gene stroma-derived prognostic predictor or 26-gene stroma-derived prognostic predictor or 26 gene* or 26-gene* or SDPP	3,255	1,911	1,344	0	2,105	12	1	1C
8-gene genomic grade index or 8 gene genomic grade index or 8-gene* or 8 gene* or GGI8	9,805	6,225	3,580	0	6,555	9	1	1C
7-gene immune response module or 7-gene immune response module or immune response module or IR7 or 7 gene* or 7-gene*	8,255	5,523	2,732	0	5,791	10	1	1C
MAGE-A or MAGEA or melanoma antigen family A	678	393	285	0	415	8	8	8C
26-gene signature or 26 gene signature or 26 gene* or 26-gene* or Novel 1 or Novel1	7,070	4,236	2,834	0	4,878	9	1	1C
B-cell:IL8 ratio or B-cell:Interleukin 8 ratio or (B-cell and Interleukin 8) or (B-cell and IL8) or Bcell signature or B-cell signature	1,551	1,276	275	0	1,513	2	2	2C
8-gene* score or 8 gene* score	7	5	2	0	4	1	1	1C
14-gene metastasis score or 14 gene metastasis score or MS14 or 14-gene* or 14 gene*	6,190	3,710	2,480	0	3,955	4	1	1C
32-gene p53 status signature or 32 gene p53 status signature or 32 gene* or 32-gene*	2,370	1,412	958	0	1,520	1	1	1C
64-gene expression signature or 64 gene expression signature or 64 gene* or 64-gene* or Pawitan	950	609	341	0	635	3	1	1C
85-gene signature or 85 gene signature or 85-gene* or 85 gene* or Iwao	694	408	286	0	421	2	1	1C
92-gene predictor or 92 gene predictor or 92-gene* or 92 gene*	729	462	267	0	466	1	1	1C
127-gene classifier or 127 gene classifier or 127-gene* or 127 gene*	360	214	146	1	219	0	0	1C

158 gene HER2-derived prognostic predictor or 158-gene HER2-derived prognostic predictor or HDPP or 158 gen* or 158-gen*	424	255	169	0	266	1	1	1C
368-gene medullary breast cancer like signature or 368 gene medullary breast cancer like signature or 368 gene* or 368 gene*	124	77	47	0	79	1	1	1C
512 gene signature or 512-gene signature or 512-gene* or 512 gene* or Olaf	229	131	98	0	158	2	1	1C
Cell cycle pathway signature or CCPs or cell cycle signature	1,188	695	493	0	769	1	1	1C
GCNs of MET or gene copy number of MET or MET GCN or MET Gene copy number	226	147	79	0	43	1	1	1C
T-cell Metagene or T cell Metagene or T cell signature or T-cell signature	194	148	46	0	143	4	1	1C
(Hormone receptor negative and triple negative) or 14 GENE* or 14-GENE*).	6,533	3,958	2,575	0	4,272	12	1	1C
(HOXB13:IL17BR or (HOXB13 and IL17BR))	98	75	23	1	94	9	7	7C
28-gen* or 28 gen*	3,302	2,037	1,265	0	2,186	1	1	1C
GeneSearch Breast Lymph Node Assay or GeneSearch or Breast Lymph Node Assay CHECK	91	62	29	1	67	10	10	2C, 5CU & 3AV
((cytokeratin-19 or cytokeratin 19 or CK-19 or CK 19) and (mammaglobin or MGB)) or METASIN	217	168	49	0	149	7	6	4C,2AV
BreastPRS or 200 gene* signature or 200 gene* algorithm	13	9	4	0	11	1	1	1C
Mammostrat or (immunohistochemical adj2 five) or IHC assay	1,470	1,070	400	0	1,024	8	6	5C, 1 CU
Breast Cancer Index or ((2-gene or HoxB13 IL17BR ratio index or HI) and (Molecular Grade Index or 5-gene microarray assay))	206	152	54	0	166	13	11	8C, 3 CU
Rotterdam gene signature or Rotterdam Signature or Rotterdam gen* or 76-gene or 76-gene or 76 gen*	600	361	239	0	379	5	4	4C
ICH4 or ICH-4 or IHC4+C or immunohistochemicaladj2 four	261	146	115	0	150	16	13	8C, 5 CU
MapQuant Dx or MapQuantDx or GGI or Genomic Grade Index or reduced Genomic Grade Index or reduced GGI or rGGI or GGI reduced or GGIr or 97-gen* or 97 gen*	1,160	718	442	0	764	21	14	10C, 4 CU
EpClin or EndoPredict or Endopredict or 11 gene* or 11-gene*	8,023	4,796	3,227	0	4,972	33	20	5C, 13 CU, 2 AV
186 gen* or invasive gene signature or IGS	7,021	3,949	3,072	0	4,370	13	1	1C
Prosigna or PAM50 or 50 GENE* or 50-GENE* ROR Score or Risk of recurrence score	5,310	3,470	1,840	0	3,555	37	35	22C, 9 CU, 4AV
MammaPrint or Mamma-Print or Mamma Print or 70 gene signature or 70 gene signature or 70-gene signature	883	674	209	0	684	87	71	34 C, 33CU, 4AV

**Oncotype DX or Oncotype-DX or Oncotype – DX or 12 gene or 21-gene or 21 – gene or recurrence score**	**7,100**	**4,791**	2,309	**0**	**5,884**	**376**	**251**	**44 C, 205 CU, 2AV**
*** In some cases CU studies addressed more than one category hence the discrepancy between the "Number of selected articles" and "Types of articles selected".**<br><br>**CL: Clinical Studies, CU: Clinical Utility Studies, AV: Analytical Validity Studies, HF: Human Factor Studies, CE: Cost Effectiveness Studies, DA: Decisional Analysis, IMPL: Implementation Studies, FEAS: Feasibility Studies**								

Additional File Table S6: Table indicating a modified version of PRISMA flow diagram. For simplicity and more effective representation of the large number of systematic searches, PRISMA flow diagram was tabulated. This tabulated PRISMA indicates details of systematic searches of 2 successful and 5 stalled breast cancer biomarkers.

	**IDENTIFICATION**				**SCREENING**	**ELIGIBILITY**	**INCLUDED**	
**Biomarker of interest**	**All Articles**	**Embase**	**Medline**	**Extra Articles**	**Records after duplicated Removal**	**Full Articles Assessed for eligibility**	**Selected Articles**	**Types of articles selected**
BRAF	4911	3588	1323	7	3909	3909	125	**51 CL, 22 AV,27 IMPL,5 FEAS,17 CU,3 CE**
KRAS	8958	6785	2173		3134	3134	139	**81Cl, 6 CE,44 CU,4 FEAS,3 IMPL,1HF**
PIK3CA	1471	1201	270	3	489	489	54	**47Cl,2AV,2CU,2 IMPL,1FEAS**
Immunoscore	531	388	143	6	960	960	12	**12Cl**
PTEN	1111	857	254	5	761	761	40	**40 Cl**
PD-L1	860	673	187	11	123	123	22	**20CL, 1CU, 1AV**
Onco-Dx	134	80	54	0	3909	3909	10	**4Cl,4 DA,1HF,1CE**
*** In some cases CU studies addressed more than one category hence the discrepancy between the "Number of selected articles" and "Types of articles selected".**<br><br>**CL: Clinical Studies, CU: Clinical Utility Studies, AV: Analytical Validity Studies, HF: Human Factor Studies, CE: Cost Effectiveness Studies, DA: Decisional Analysis, IMPL: Implementation Studies, FEAS: Feasibility Studies**								

Additional File Table S7: Detailed Attributes extracted from systematic literature and guidelines.			
**MAIN CATEGORY**	**ATTRIBUTE DETAIL**	**SUB-ATTRIBUTE CATEGORY**	**REFERENCE**
**RATIONALE**	Identify the unmet clinical need for a biomarker	*Unmet need*	Monaghan et al., 2018; Taube 2009
	Verify the unmet need for the biomarker - is there an existing solution?	*Verification of unmet Need*	Taube et al. 2009; CONSORT 2010; STROBE; ARRIVE; Conley & Taube 2004; SQUIRE
	Study states the pre-specified hypothesis	*Pre-specified hypothesis*	Sauerbrei et al., 2014; REMARK CONSORT (2010) STROBE STARD
	BM type: **Screening/ Diagnostic BMs**	*BM type*	Pavlou et al., 2013; Silva 2015; Cho 2007; Baker 2009; Hendriks et al.;2017
	**Predictive BMs**		Rodrigues-Enriques et al., 2011; Ellis et al.,2011, Harris et al.,2007; Landgren & Morgan 2014; Kalia 2015; Merrer & Dieterle 2008; Taube 2009; Montie & Meyers 1997; Fertig & Hayes 2001; Schneider et al., 2015; Conley & Taube 2004
	**Pharmacodynamic BMs**		Modur et al., 2013; Merrer & Dieterle 2008
	**Response BMs**		Modur et al., 2013
	**Prognostic BMs**		Ellis et al., 2011; Ocker (2018) Juarez-Hernandez et al., 2017; Seregni et al., 2004; Baker 2009; Conley & Taube 2004; Yang et al., 2019; Kalia 2015; Pollack et al., 1998; Sturgeon 2010; Pavlou et al., 2013; Silva 2015; Cho 2007; Baker 2009;

			Pollack et al. 1998; Sturrgeon 2010; Volpe et al., 2018; Montie & Meyers 1997; Harris et al., 2007; Volpe et al., 2018 Merrer & Dieterle (2008) Pavlou et al. (2013) Juarez-Hernandez et al. (2017) Juarez-Hernandez et al. (2017) Sauerbrei et al. (2014) – REMARK Pepe et al. (2008) - PROBE
**ANALYTICAL VALIDITY**	Was the sample collected from the organ(s) of origin / was the biospecimen obtained from diseased section? (If sample was obtained from a distal source or adjacent, e.g. blood, score 0).	*Anatomical or collection site*	Gromov et al., 2014 ; BRISQ
	Is the proximity to primary pathology of interest stated?	*Anatomical or collection site*	Gromov et al., 2014 ; BRISQ
	Study acknowledges noncompliance (deviation from protocol)	*Assay Validation*	Baker. 2009
	Study adjusts for post screening noise	*Assay Validation*	Baker 2009; Pepe et al., 2015 & Ewaisha et al., 2015
	Is biomaker analyte linear on dilution? Analyte recovery should also be documented	*Assay Validation*	Hayes et al., 1996; T.W., N.E., & J.D., 2001; Kensler et al., 2001; Cumminget et al., 2008; Sturgeon et al 2010
	Is the technique quality assured (i.e., is it a commercially available assay kit, or a widely known/used techqniue)?	*Assay Validation*	Sauerbrei et al., 2014; REMARK
	Are the biomarker test results reproducible: is the biomarker test repeated in duplicates/triplicates for each specimen?	*Assay Validation*	Taube et al., 2009; Tan et al., 2009; Helzlsouer 1994; Boutros 2015; Hayes et al., 1996; Feng, Kagan, Pepe, Thornquist, Ann Rinaudo, et al., 2013; Fuzery et al., 2013 Cummings et al., 2008; Hristova & Chan, 2019; Zhang & Chan 2010; Pavlou et al. 2013; Duffy & Sturgeon 2015; Conley & Taube 2004; Sauerbrei et al. 2014; REMARK Miquel-Cases et al. 2017; Hayes et al., 2013

	Is the study repeatable; was the biomarker tested in different laboratories?	*Assay Validation*	Taube et al., 2009; Tan et al., 2009; Helzlsouer 1994; Boutros 2015, Zhang & Chan 2010; Pavlou et al., 2013; Duffy & Sturgeon 2015; Conley & Taube 2004; Sauerbrei et al., 2014, REMARK, Miquel-Cases et al., 2017; Hayes et al., 2013
	Is the level of biomarker biological noise/background tested (i.e., is the influence of biomarker cross-reactivity or carry over addressed in the methodology?)	*Assay Validation*	Sauerbrei et al., 2014; REMARK; STARD; Tockman et al., 1992; Hammond & Taube 2002; Paulovich et al., 2008.
	Does the biomarker assessment methodology include use of calibration curves to define analyte concentration?	*Assay Validation*	Cummings et al., 2008; George, 2008; Chau et al., 2009; Fuzery et al., 2013
	Analytical sensitivity-has the limit of detection for the biomarker been stated?	*Assay Validation*	Hayes et al., 1996; Cummings et al., 2008; Chau et al., 2009; Daidone et al., 2011; Wagner & Srivastava, 2012c; Fuzeri et al., 2013; Mordente et al., 2015; Salgado et al., 2017; Hristova & Chan, 2019
	Does the biomarker assay consider the degree of analytical variation, e.g. does it take into consideration the influence of unrelated matrix components?	*Assay Validation*	
	Does the study include methods to understand biomarker variability, e.g. does it include the effects of time as variable?	*Assay Validation*	TRIPOD
	Is the variability of biomarker measurement addressed, e.g. does the study evaluate coefficient of variation?	*Assay Validation*	Wagner & Srivastava, 2012c; Weber et al., 2012a; Bossuyt et al., 2003; Mcshane et al., 2005; Cummings et al., 2008; George, 2008; Paulovitch et al.,, 2008; Chau et al., 2009; Viale et al., 2009; Sturgeon et al., 2010; Fuzery et al., 2013;Network, 2016;Salgado et al., 2017;Miquel-cases et al., 2017
	Are the reagents used quality assured, i.e., from a commercial seller?	*Assay Validation*	Sauerbrei et al., 2014; REMARK

	Does the study specify the assay method/technique used?	*Assay Validation*	Conley & Taube., 2004; Sauerbrei et al., 2014; REMARK
	Is the biomarker study validated/standardised/optimised?	*Assay Validation*	Hammond & Taube, 2002; Taube et al., 2009; Schneider et al., 2015; Modur et al., 2013; Merrer & Dieterle. 2008; Hayes 2013; Sargent & Allegra 2002; Montie & Meyers 1997; Conley & Taube 2004
	Was the sample collected using a standardised protocol (SOP-Standard Operating Procedure)?	*Biospecimen Collection Technique*	Gion & Fabricio 2018;King et al., 2014; Duffy & Sturgeon 2015; Pavlou et al., 2013; Ewaisha et al., 2015; Hritsova & Chan 2019;Hayes 2013; Maes 2015; Pepe et al., 2008;PROBE; Hammond & Taube 2002; CONSORT 2010; Baker 2009; Wang 2014
	Does the study specify detailed procedures for specimen collection (e.g. whether samples were collected before or after study question was set, were collected from patients with refractory disease or at time of relapse or were collected when patient was dead or alive)?	*Biospecimen Collection Technique*	Pepe et al., 2008; PROBE; Hammond & Taube 2002; CONSORT 2010;Baker 2009; BRISQ; Rimza et al. 2016; Costello et al., 2011
	Is the method of biospecimen attainment stated (e.g., fine needle aspiration, pre-operative blood draw)?	*Biospecimen Collection Technique*	BRISQ
	Is the collection container of the biospecimen stated?	*Biospecimen Collection Technique*	BRISQ
	Is the size or weight of solid biospecimen samples being processed clearly stated (e.g., cubes approximately 0.5 cm on a side, 0.5 gram)?	*Biospecimen Collection Technique*	BRISQ
	Are the inclusion/exclusion criteria of the biomarker stated (e.g., a minimum threshold for DNA, minimum amount of tumour cells in the sample)?	Biospecimen Inclusion/Exclusion Criteria	Mordente et al., 2015 & BRISQ
	Is the specimen condition is described, e.g. frozen, fresh, primary, metastatic?	*Biospecimen matrix/type*	Hammond & Taube 2002
	Is the specimen described as solid tissue, whole blood or serum/plasma/isolated cells?	*Biospecimen matrix/type*	Sauerbrei et al. 2014; REMARK
	If applicable, are cell culture details described?	*Cell Culture*	
	Do the authors mention sample stability?	*Biospecimen Quality*	BRISQ

	Description of storage; is the sample stored stably (e.g., stated frozen temperature or fixed)?	*Biospecimen Quality*	BRISQ
	Are cycles of freeze and thaw described?	*Biospecimen Quality*	BRISQ
	If animals used, is the following defined: species, strain, sex, source, genotype, immune status, developmental stage and weight?	*Experimental animals*	ARRIVE
	Is the relevant health status of animals before treatment or testing reported (e.g. weight, microbiological status, and drug or test naïve)?	*Experimental animals*	ARRIVE
	Are details of experimental work clearly explained to allow experimental replication?	*Experimental Procedure Description*	ARRIVE & STROBE
	Is the biospecimen processing described, e.g., was the specimen snap frozen, controlled-rate freezed, heparin/citrate/EDTA fixed?	*Mechanism of stabilization/*	BRISQ
	If frozen, is the temperature of biospecimen freezing stated?	*Mechanism of stabilization/*	BRISQ
	Is the constitution and concentration of fixative stated?	*Mechanism of stabilization/*	BRISQ
	Is the biospecimen processing timing described, e.g., is the time in fixative/preservation solution stated?	*Mechanism of stabilization/*	BRISQ
	Is the biospecimen method of enrichment stated, e.g., do the authors state that laser-capture microdissection of tissue/block selection for region of lesion/ centrifugation of blood etc. were used to enrich the specimen prior to analysis?	*Sample Pre-processing*	BRISQ
	Were biospecimen quality-assurance measures applied, e.g., was the RNA of the specimen assessed prior/after long-term storage and immediately before experimental analysis?	*Sample Pre-processing*	BRISQ;Rimza et al.,2016
	Is the storage temperature described?	*Storage/Shipping /Transport*	BRISQ
	Is the storage duration described?	*Storage/Shipping /Transport*	BRISQ
	Are storage details described?	*Storage/Shipping /Transport*	BRISQ
	Are the shipping parameters stated, e.g., vacuum sealing, desiccant, packing material etc. ?	*Storage/Shipping /Transport*	BRISQ
	Is shipping temperature (s) stated?	*Storage/Shipping /Transport*	BRISQ
	Is shipping duration described?	*Storage/Shipping /Transport*	BRISQ
	Is the type of transport container described?	*Storage/Shipping /Transport*	BRISQ
	Is the number of freeze-thaw cycles described?	*Storage/Shipping /Transport*	BRISQ
	Is the duration of thaw events described?	*Storage/Shipping /Transport*	BRISQ

	Time from last thaw to processing described?	*Storage/Shipping /Transport*	BRISQ
	Temperature between last thaw and processing described?	*Storage/Shipping /Transport*	BRISQ
	Does the time or range of time between disease diagnosis and sample acquisition affect bio specimen quality?	*Time between diagnosis and sampling*	STARD
	Was the biospecimen collected when the patient was alive (Y) or deceased?	*Vital state of Biospecimen*	STARD
**CLINICAL VALIDITY**	Does the study mention factors associated with their sample collection (such as fasting status, posture, circadian rhythms, age and sex) and do they investigate their relation to the analyte of interest?	*Analytical modelling*	Pavlou et al., 2013; Sauerbrei et al., 2014 & REMARK
	Model performance: Define all predictors used in developing or validating the multivariable prediction model, including how and when they were measured	*Analytical modelling*	TRIPOD; Sauerbrei et al., 2014; REMARK; STARD
	Model Specification: do the authors present the full prediction model to allow predictions for individuals? Do they mention regression coefficients/confidence intervals/ p values/ baseline survival at a given time point?	*Analytical Modelling*	TRIPOD; Sauerbrei et al., 2014; REMARK; STARD
	**Model-updating**: If done, report the results from any model updating (i.e., model specification, model performance)	*Analytical Modelling*	SPIRIT
	Were the scientists analysing the biomarker results blinded to the clinical outcome of patients, and vice-versa?	*Blinding*	Pepe et al., 2008; PROBE; Sauerbrei et al., 2014; REMARK; PROBE
	Are outcomes reported with precision (e.g. standard error or confidence interval)?	*Experimental Outcomes*	STROBE;Duffy & Sturgeon 2015
	Does the index test answer the review question?	*Experimental Outcomes*	Pepe et al., 2008; PROBE; CONSORT 2010; STROBE; Taube et al., 2009; Sauerbrei et al., 2014; REMARK
	How are the biomarker end-points determined: what cut-off or threshold will be used to distinguish positive or negative outcomes?	*Experimental Outcomes*	Costello et al., 2011; Pepe et al., 2008; PROBE/Sauerbrei et al., 2014; REMARK; STARD Tockman et al., 1992; Hammond & Taube 2002; Paulovich et al., 2008.
	Were outcomes reported with precision, e.g. clearly stated with 95% confidence level and effect size?	*Experimental Outcomes*	STROBE;Duffy & Sturgeon 2015
	Is the data presented as an absolute value as well as relative effect size? (Both are needed to score 1)	*Experimental Outcomes*	CONSORT 2010; Sauerbrei et al., 2014; REMARK
	Is the study externally validated in a separate cohort?	External Validation	Diamandis 2012; Bast et al., 2005; Schneider et al.,

			2015;<br>Campbell 2016; Hayes et al. 1996; TMUGS; Taube 2009;<br>Shirodkar & Lokeshwar 2008;<br>Taube et al., 2005;<br>Sauerbrei et al., 2014;<br>REMARK; Merrer & Dieterle 2008
	If relevant, does the study give details of treatments received (including type and timings of chemotherapy courses)?	*Intervention*	CONSORT 2010; STARD;<br>Sauerbrei et al., 2014;<br>REMARK
	Are the interventions for each group described with sufficient details to allow replication, including how and when they were actually administered?	*Intervention*	CONSORT 2010; STARD;<br>Sauerbrei et al., 2014;<br>REMARK
	If present, are changes to the methodology clearly stated in the protocol, e.g., changes in eligibility criteria, with reasoning?	*Methodology Details*	CONSORT 2010
	Is the handling of missing data described?	*Missing Data*	Sauerbrei et al., 2014;<br>REMARK; STROBE;<br>STARD;<br>SQUIRE; Taube 2009;<br>Panis et al., 2016; ARRIVE
	Does the study include the participants medical history, including medication and additional disease that might affect the biospecimen?	*Patient Confounding Factors*	Pepe et al., 2008; PROBE;<br>Sauerbrei et al., 2014;<br>REMARK; CONSORT (2010); STROBE; STARD
	Are the eligibility criteria clearly stated, e.g., symptoms, previous test results and inclusion registry?	*Patient Eligibility*	STROBE
	Exclusion Criteria- Did the study avoid inappropriate exclusions?	*Patient Eligibility*	Pepe et al., 2008; PROBE;<br>Sauerbrei et al., 2014;<br>REMARK; CONSORT (2010); STROBE; STARD
	Is the flow of participants through the study described, including the number of participants with and without the outcome and, if applicable, a summary of the follow-up time?	*Patient Eligibility*	Sauerbrei et al.,2014;<br>REMARK; CONSORT (2010); STROBE; STARD
	Are the characteristics of the participants described (basic demographics, clinical features, available predictors), including the number of participants with missing data for predictors and outcome?	*Patient Eligibility*	Sauerbrei et al. 2014;<br>REMARK
	Is the setting, location and dates of eligible patients stated?	*Patient Eligibility*	CONSORT 2010; STARD;<br>AGREE 2016; Sauerbrei et al., 2014; REMARK;<br>STROBE; Ewaisha et al., 2015; Pepe et al., 2008;<br>PROBE; Hammond &

			Taube 2002; CONSORT 2010; Baker 2009; Hayes 2013
	Are the characteristics described for the base case population and subgroups analysed, including why they were chosen (histopathologic data, demographics etc.)?	*Patient Eligibility*	Panis et al., 2016; Pepe et al., 2008; PROBE; Sauerbrei et al., 2014; REMARK; CONSORT 2010; STROBE; STARD; AGREE 2016; CHEERS; Maes 2015
	Does the study match control subjects to case patients on suitable factors and describe matching criteria and number of exposed/unexposed?	*Patient Eligibility*	Pepe et al., 2008; PROBE STROBE
	Does the study describe distribution of disease severity in the cases (e.g. tumour stage)?	*Patient Eligibility*	STARD; AGREE 2016
	Does the study interpret the results in the context of the pre-specified hypotheses and other relevant studies (i.e. pilot data)?	Pre-specified hypothesis	Duffy & Sturgeon 2015; Paulovich et al., 2008
	Does the study reference, or is a pilot study that has identified, the optimal sample collection and storage condition? Or, does the study include or use pilot measurements of biomarker's performance characteristics in the desired clinical setting?	Pre-specified hypothesis	Sauerbrei et al.,2014; REMARK
	Is the population randomised?	*Randomisation/Blinding*	Pepe et al., 2008;PROBE; Maes 2015
	Is the method used to generate the random allocation sequence stated?	*Randomisation/Blinding*	CONSORT 2010; ARRIVE
	Type of randomisation; are details of any restriction are clearly stated?	*Randomisation/Blinding*	CONSORT 2010; ARRIVE
	Is it stated who generated the random allocation sequence, enrolled participants, and assigned participants to interventions?	*Randomisation/Blinding*	CONSORT 2010
	Did all patients receive a reference standard (i.e., the equivalent gold standard test, if available)?	*Reference Standard*	CONSORT
	Did patients receive the same reference standard?	*Reference Standard*	Wang 2014
	Was the interval between index test and reference standard stated, and if so, was the index test conducted within a reasonable time from the reference standard?	*Reference Standard*	QUADAS2; Bossuyt et al., 2003; Maria Grazia Daidone, Nadia Zaffaroni, Vera Cappelletti; Wagner & Srivastava, 2012c; Mordente et al., 2008;Hristova & Chan, 2019
	Does the study use a reference standard, to assess outcome?	*Reference Standard*	CONSORT
	Is there explanation of the choice of sample size, for example, was it based on pilot data, or did the authors use a power calculation?	*Sample size Calculation*	Pepe et al., 2008; PROBE; Pavlou et al. 2013; ARRIVE

			Sauerbrei et al., 2014; REMARK; STROBE; STARD Baker 2009; Pepe et al., 2015; Conley & Taube 2004; Zolg 2006; Hritsova & Chan 2019; Costello et al.,2011;Conley & Taube 2004; Maes 2015
	Are details of sample size calculation stated?	*Sample size Calculation*	Hammond & Taube (2002)
	Were the methods for estimating or comparing measures of diagnostic accuracy stated? (Positive Predictive Value, Negative Predictive Value, Survival)	*Sensitivity/ specificity*	Cummings et al., 2008; George et al.,2008; Chau et al., 2009; Fuzery et al., 2013; Duffy & Sturgeon 2015; Pepe et al., 2008; PROBE;Chen et al., 2018; Riechl & Mikultis 2016; Volpe et al., 2018; Juarez-Hernandez et al., 2017;Shirodkar & Lokeshwar 2008; Hendriks et al., 2017; Silva 2015; Seregni et al., 2004; Tockman et al., 1992; Cho 2007; Montie & Meyers 1997; Locke et al., 2019; Schneider et al., 2015; Maruvada & Srivastava 2006; Poste et al., 2012; Donovan & Cordon-cardo 2013; Conley & Taube 2004; Helzlsouer 1994; Kvinnsland 1991; Diamandis 2012; Nicollete & sMiller 2003; Negm et al.,2002; Bast et al., 2005; Paulovich et al., 2008; Handy 2009; Pavlou et al., 2013; Maes 2015; Ali et al., 2018; Wang 2014; Yang et al., 2019; Landgren & Morgan 2014; Riechl & Mikultis 2016; Wentzensen et al., 2013; Baker 2009; Bast et al., 2005; Handy 2009; STARD

	Was specificity and sensitivity stated?	*Sensitivity/ specificity*	Cummings et al., 2008; George et al.,2008; Chau et al., 2009; Fuzery et al., 2013; Duffy & Sturgeon 2015; Pepe et al., 2008; PROBE;Chen et al., 2018; Riechl & Mikultis 2016; Volpe et al., 2018; Juarez-Hernandez et al., 2017;Shirodkar & Lokeshwar 2008; Hendriks et al., 2017; Silva 2015; Seregni et al., 2004; Tockman et al., 1992; Cho 2007; Montie & Meyers 1997; Locke et al., 2019; Schneider et al., 2015; Maruvada & Srivastava 2006; Poste et al., 2012; Donovan & Cordon-cardo 2013; Conley & Taube 2004; Helzlsouer 1994; Kvinnsland 1991; Diamandis 2012; Nicollete & sMiller 2003; Negm et al.,2002; Bast et al., 2005; Paulovich et al., 2008; Handy 2009; Pavlou et al., 2013; Maes 2015; Ali et al., 2018; Wang 2014; Yang et al., 2019; Landgren & Morgan 2014; Riechl & Mikultis 2016; Wentzensen et al., 2013; Baker 2009; Bast et al., 2005; Handy 2009; STARD
	Was the study designed to detect a specified effect size? Does the study give target power and effect size?	*Statistical Modelling*	Taube et al., 2009; Handy 2009; Sauerbrei et al. 2014; REMARK
	Did the authors recalibrate their initial model, upon study validation?	*Statistical Modelling*	SPIRIT
	Does the study describe and give reasons for the specific type of decisional analytical model used? (Providing a figure to show model structure is strongly recommended)	*Statistical Modelling*	REMARK; SQUIRE; SPIRIT
	Among reported results, does the study provide estimated effects, with confidence intervals?	*Statistical Modelling*	REMARK

	Does the study present univariable analyses showing the relation between the marker and outcome, with the estimated effect (e.g., hazard ratio and survival probability)? Does it provide similar analyses for all other variables being analysed? (For the effect of a tumour marker on a time-to-event outcome, a Kaplan-Meier plot is recommended.)	*Statistical Modelling*	Sauerbrei et al. (2014) - REMARK
	For key multivariable analyses, are estimated effects (e.g., hazard ratio) reported with confidence intervals for the marker and, at least for the final model, all other variables in the model?	*Statistical Modelling*	STARD
	If relevant, do the authors describe the reasons for the decisional analysis model used?	*Statistical Modelling*	REMARK; SQUIRE; SPIRIT
	Do the authors describe over-fitting data/variables, or subjectively are there too many variables?	*Statistical Modelling*	George et al., 2008
	Does the study use appropriate quality controls for statistical analysis, e.g., have the authors collaborated with an experienced biostatistician?	*Statistical Modelling*	Pavlou et al., 2013; Ewaisha et al., 2015
	Does the study present a summary of trial design (including allocation ratio/methods/results/conclusions), states registration number and name of trial registry and where the full trial protocol can be accessed, if available?	*Trial Design description*	CONSORT 2010; ARRIVE; CONSORT 2010; STARD
	Did the authors explain all important adverse events in the study? Have they explained modifications to the experimental protocol upon study commencement?	*Adverse events*	ARRIVE<br>CONSORT (2010)
**CLINICAL UTILITY**	Does the biomarker have approval for clinical use (e.g. from NICE or FDA)?	*Authority Approval*	Costello et al., 2011; Hayes 2013; Pepe et al., 2015; Ewaisha et al., 2015
	Subjectively, might this biomarker result in cost-saving changes to clinical practice such as reduced hospital admissions, reduced chemotherapy or a reduction in more expensive diagnostic tests/treatments?	*Cost-effectiveness*	CHEERS; Taube et al., 2009
	Does the study discuss costs and strategic trade-offs (including opportunity costs)?	*Cost-effectiveness*	Taube et al., 2009; Wang 2014;<br>Shirodkar & Lokeshwar 2008;<br>Hendriks et al., 2017;<br>Locke et al., 2019;<br>Schneider et al., 2015;<br>Helzlsouer 1994; Negm et al., 2002; Handy 2009;<br>Yang et al., 2019;<br>Monaghan et al., 2018;<br>CHEERS

	Decisional Analysis- subjectively, might the biomarker influence clinician decision making?	*Decisional Analysis*	Poste et al., 2012; Hayes et al., 1996; TMUGS; Taube 2009; Shirodkar & Lokeshwar 2008; Taube et al., 2005, Sauerbrei et al., 2014; REMARK, Sauerbrei et al., 2014
	If relevant, does the study include ethical review permissions, relevant licences for in vivo animal work (e.g., Animal [Scientific Procedures] Act 1986), and national or institutional guidelines for the care and use of animals, that cover the research? Ethics for patient sampling and any interventions should also be clearly stated.	*Ethics*	ARRIVE &
	Can the biomarker be incorporated in routine care workflow / can it be implemented in clinical practice?	*Feasibility*	Hammond & Taube 2002; Taube 2009; Wang 2014
	Does it involve High-Throughput techniques?	*Feasibility*	Paulovitch et al., 2008; Sturgeon et al., 2010; Hritsova & Chan 2019; Rimza et al., 2016; Modur et al., 2013; Helzlsouer 1994
	Is the biomarker assay automated? (If it requires a lot of work force, score 0)	*Feasibility*	SQUIRE
	Does the study state sources of funding and other support (such as supply of drugs), role of funders and provide an explicit statement that all group members have declared whether they have competing interest?	Funding	CONSORT (2010); STROBE;STARD; AGREE (2016); ARRIVE; AGREE (2016); CHEERS
	Are the all-important harms or unintended effects in each group are stated?	*Harms and Toxicology*	Miquel-Cases et al., 2017; Negm et al., 2002
	If relevant, is the toxicology of the biomarker target being tested explained?	*Harms and Toxicology*	SQUIRE
	Are Human Factors, such as the invasiveness of sample collection or acceptance of the test by clinicians, considered or discussed?	*Human Factor*	STARD, George, 2008; Hristova & Chan, 2019; Pollack et al., 1998; Sturgeon 2010
	Was sample collection non-invasive?	*Invasiveness*	Helzlsouer 1994; Silva 2015; Wang 2014;Tan et al., 2009; Shirodkar & Lokeshwar 2008
	Were specimens collected prospectively?	*Study Type*	Poste et al., 2012; Pepe et al., 2008; PROBE; Hammond & Taube 2002; CONSORT 2010; Baker 2009; Hayes 2013, STARD

	Does the study acknowledge limitations, e.g., does it take into consideration benefits/ harms/study limitations?	*Utility*	CONSORT 2010; ARRIVE; CONSORT 2010; STARD
	Is there discussion regarding who will benefit from the biomarker, what the intended utility of biomarkers is and/or whether it can be used on both high and low income individuals?	*Utility*	STARD (intro); Celis et al., 2005;George 2008; Chau et al., 2009; Sturgeon et al., 2010; Wagner & Srivastava, 2012c; Daniel F Hayes, 2013;Campbell, 2016; Miquel-Cases et al., 2017; Salgado et al., 2017;Hristova & Chan, 2019
	Can the findings of this study be translated to other species including humans?	*Utility*	Schneider et al., 2015; STARD<br>Taube et al., 2009; Hayes 2014;<br>Hendriks et al., 2017; Locke et al., 2019; Handy 2009;<br>Pavlou et al., 2013; Taube 2009; Sargent & Allegra 2002;<br>Tockman et al., 1992; Sauerbrei et al., 2014; REMARK; Negm et al., 2002
	Does the study define a specific algorithm to assess biomarker outcome, in addition to other information, including clinical information and other avialable markers etc?	*Utility*	Schneider et al., 2015; STARD<br>Taube et al., 2009; Hayes 2014;<br>Hendriks et al., 2017; Locke et al., 2019; Handy 2009;<br>Pavlou et al., 2013; Taube 2009; Sargent & Allegra 2002;<br>Tockman et al., 1992; Sauerbrei et al., 2014; REMARK; Negm et al., 2003
	Is the biomarker linked with a current health policy/health practice?	Utility	AGREE 2016; Poste et al., 2012
	Regarding the results and discussion, are observed associations between outcomes, interventions, and relevant contextual elements clearly stated? Are unintended consequences such as unexpected benefits, problems, failures, or costs associated with the intervention(s) reported?	*Utility*	CONSORT

	Is biospecmin collection amenable to pre and post- operative treatment sampling? (e.g. Biopsy no, breath/blood yes)	*Utility*	G.F., J., H., T., & A., 2012
	Does the study specify the time period from which cases were taken and/or specifies median/end of follow-up period?	Utility	Sauerbrei et al., 2014; REMARK; CONSORT 2010; STROBE
	Does the study evaluate increment in performance when biomarker is combined with current relevant methods?	Utility	Pepe et al., 2008; PROBE
	Does the study state if samples were obtained and processed in a way similar to what will occur in a clinical setting?	*Utility*	Poste et al., 2012; STTaube et al., 2009; Sauerbrei et al., 2014; REMARK; CONSORT 2010; STROBE; STARD; ARRIVE; CHEERS; Baker (2009); STROBE;ARRIVEROBE; STARD; CHEERS
	Does the study address if the biomarker use can be beneficial outside the clinical trial setting or does the study address if the biomarker results can be generalised outside a clinical trial?	*Utility*	Poste et al., 2012; STTaube et al., 2009; Sauerbrei et al., 2014; REMARK; CONSORT 2010; STROBE; STARD; ARRIVE; CHEERS; Baker (2009); STROBE;ARRIVE; STARD; CHEERS

Additional File Table S8a: Result Summary of stage A- round 1 online Delphi Survey		
**Attribute Category**	**Number of Attribute-groups assessed (n) ***	**% of attribute-groups in which <75% consensus was achieved (n)**
Analytical Validity	13	92.31 (12)
Clinical Validity	16	93.75 (15)
Clinical Utility	17	70.59 (12)
Rationale	5	80 (4)
**All Categories**	**51**	**84.31 (43)**
*Detailed Attributes are found in **additional file: Table S7**. Attributes were grouped according to theme to simplify the questions and allow the participants to answer the question more efficiently.		

Additional File Table S8b: Table indicating characteristics that moved into the second round of the Delphi Survey, and the % agreement reached in round 1 and 2, respectively			
**Category**	**Characteristics**	**Round 1**	**Round 2**
AV	Detailed description of experimental animals if used (i.e. strain, sex, weight & relevant health status)	66.67	66.67
CV	Randomisation: Is the population randomised and in what way? How is the random allocation generated?	70.59	80.56
CU	Can the biomarker result be delivered via machine learning?	43.16	25
CU	Scalability: High Throughput technique	66.67	80.56
CU	Can the findings of the study be translated to other species including humans?	64.71	47.22
CU	Affordability for the patient. Is there a reimbursement?	47.06	36.11
CU	Can the biomarker be applied to assess the health of a close family member?	43.14	30.56
Rationale	Applicable to a wide cohort	70.59	63.89

Additional File Table S9: Table showing the attribute-categories identified as significantly different between successful and stalled breast cancer biomarkers, using Man Whitney- U test and binary logistic regression*

	Successful		Stalled		Mann-Whitney		Binary Logistic Regression (95% C.I.)		
	**Mean**	**SEM**	**Mean**	**SEM**	**P-Value**	**P value Summary**	**Sig.**	**Lower**	**Upper**
**Adverse events**	**33.33**	**4.62**	**17.07**	**4.18**	**0.02**	*	**0.01**	**0.98**	**1.00**
**Assay Validation- Variability/%CV**	**49.44**	**1.89**	**34.44**	**1.39**	**<0.0001**	****	**0.000**	**0.94**	**0.97**
**Assay Validation- Method Optimisation**	**47.87**	**5.18**	**34.78**	**4.07**	**0.046**	*	**0.047**	**0.99**	**1**
**Biospecimen Inclusion/Exclusion Criteria**	**86.67**	**3.33**	**54.88**	**5.53**	**<0.0001**	****	**0.00**	**0.98**	**0.99**
**Methodology Details**	**32.38**	**4.59**	**19.51**	**4.40**	**0.07**	**NS**	**0.05**	**0.99**	**1.00**
**Patient Eligibility**	**75.77**	**1.74**	**57.99**	**2.91**	**<0.0001**	****	**0.00**	**0.95**	**0.98**
**Randomisation/Blinding**	**15.48**	**2.82**	**4.573**	**1.38**	**0.01**	**	**0.00**	**0.96**	**0.99**
**Reference Standard**	**13.57**	**2.14**	**19.51**	**2.46**	**0.02**	*	**0.05**	**1.00**	**1.03**

*32 Binary Regression Analysis were conducted. The total sub-categories were 48. We excluded: i) details of experimental animal reporting which was removed from the Delphi Round 2 (n=1) i) rationale related sub-attributes (n=4), ii) Clinical Utility attributes prior Clinical Utility score amendment methodology (n=11_see **additional file: methods**).

Additional File Table S10: Table showing attribute-categories identified as significantly different between successful and stalled CRC biomarkers, using Man Whitney- U test and binary logistic regression

	Successful		Stalled		Mann Whitney U test		Binary logistic Regression		
							Sig.	95% C.I.for EXP(B)	
	Average (%)	STDEV	Average	STDEV	P-Value			Lower	Upper
**Adverse events**	56.39	4.31	35.51	4.30	0.0006	***	0.001	0.987	0.996
**Assay Validation (non-compliance)**	30.16	1.83	23.78	1.48	0.0271	*	0.008	0.971	0.996
**Assay Validation**	83.46	3.23	62.32	4.14	<0.0001	****	0.000	0.983	0.995
**Biospecimen Inclusion/Exclusion Criteria**	60.90	4.25	47.10	4.26	0.023	*	0.023	0.990	0.999
**Cell Culture**	0.75	0.75	14.25	2.11	<0.0001	****	0.000	1.034	1.105
**Experimental Procedure Description**	97.74	1.29	91.30	2.41	0.0208	*	0.031	0.973	0.999
**Harms and Toxicology**	58.27	3.62	30.07	3.19	<0.0001	****	0.000	0.977	0.989
**Intervention**	95.49	1.73	64.49	3.82	<0.0001	****	0.000	0.962	0.982
**Mechanism of stabilization/**	35.96	2.18	39.31	2.74	<0.0001	****	0.000	1.010	1.035
**Patient Eligibility**	61.07	4.25	41.10	4.26	0.001	***	0.001	0.967	0.991
**Reference Standard**	18.42	2.69	7.07	1.61	0.0007	***	0.001	0.972	0.992

*****32 Binary Regression Analysis were conducted. The total sub-categories were 48. We excluded: i) details of experimental animal reporting which was removed from the Delphi Round 2 (n=1) i) rationale related sub-attributes (n=4), ii) Clinical Utility attributes prior Clinical Utility score amendment methodology (n=11_see **Additional file: methods**).

Additional File Table S11: Table indicating the median rank for each subcategory (1-5), in Analytical Validity, Clinical Validity, Clinical Utility and Rationale for the seven indicated biomarker types (n=7).

		Diagnostic Biomarker	Response Biomarker	Predictive Biomarker	Recurrence Biomarker	Therapeutic Biomarker	Safety Biomarker	Pharmacodynamic Biomarker
ANALYTICAL VALIITY	1) Assay validation	1	1	1	1	1	1	1
	2) Detailed description of experimental animals	4	4	4	5	4	4	4
	3) Detailed description of biospecimen storage & shipping	3	3	4	3.5	3	3	3
	4) Detailed description of biospecimen source and collection	2	2	3	2	2	3	3
	5) Details of sample-pre processing	3	3	3	2	2	3	3
CLINICAL VALIITY	1) Participant eligibility	2	2	2	2	2	2	3
	2) Experimental Outcomes, adverse events, missing data or modifications to experimental protocol	4	4	4	3	3	3	3
	3) Analysis: Were the methods for estimating or comparing measures of diagnostic accuracy stated	2.5	2	3	3	3	3	3
	4) Experimental design: i.e. appropriate reference standard, sample size calculation etc	2	2	2	2	2	2	2
	5) Statistical analysis and Analytical Modelling	3	3	3	3	3	3	3
CLINICAL UTILITY	1) Usefulness/ Impact of the project on people and systems	2	1.5	2	2	2	2	2.5
	2) Regulatory Authority/Ethical Approval & Harms and Toxicology	4	4	4	4	4	3	3
	3) Human Factors	3	3	3	3	3	3	3
	4) Cost effective for both hospital and patients	3.5	4	4	4	4	4	4
	5) Can the test be easily adopted in a clinical setting?	2	2	2	2	2	2	2
RATIONALE	1) Identification of a disease of unmet need	1.5	3	2	3	2	3	3
	2) Is there an existing biomarker test in current practice? Is there a need for an improved biomarker test?	3	3	2.5	2	2	3	3
	3) Exploratory or hypothesis driven biomarker discovery approach	4	3	3	3.5	4	3	3
	4) Applicable to a wide cohort	3	3	2	3	3	2	2
	5) Identification of a biomarker type which is most useful for the disease of interest	2	2	2	2	2	2	2

Additional File Table S12: **Cox Regression Model for unweighted, weighted, and weighted top 3 categories, Breast Cancer Biomarker scores.**

		SE	Sig.	Exp(B)	95.0% CI for Exp(B)	
					Lower	Upper
Unweighted	CV1	0.007	<0.00	0.959	0.945	0.973
	CV2	0.008	0.845	0.998	0.983	1.014
	CV3	0.008	0.87	1.014	0.998	1.029
	CV4	0.014	0.669	0.994	0.967	1.022
	CV5	0.011	0.068	1.021	0.998	1.043
	CU1	0.16	0.957	0.999	0.969	1.031
	CU2	0.007	<0.000	0.966	0.952	0.98
	CU3	0.014	0.209	1.017	0.99	1.045
	CU4	0.006	0.234	0.993	0.982	1.005
	CU5	0.021	0.85	1.004	0.963	1.046
	AV1	0.13	0.025	0.972	0.948	0.996
	AV2	0.021	0.052	1.043	1	1.087
	AV3	0.014	0.105	1.023	0.995	1.053
	AV4	0.015	0.217	0.982	0.954	1.011
	AV5	0.009	0.376	0.992	0.976	1.009
	AV	0.033	0.578	0.981	0.919	1.048
	CV	0.033	0.714	1.012	0.949	1.079
	Amended CU	0.028	0.039	0.943	0.893	0.997
	TS	0.19	>0.000	0.901	0.869	0.935
Weighted-All	CV1	0.009	<0.000	0.95	0.933	0.967
	CV2	0.015	0.462	1.011	0.981	1.042
	CV3	0.011	0.136	1.017	0.995	1.04
	CV4	0.018	0.656	0.992	0.957	1.028
	CV5	0.018	0.08	1.031	0.996	1.068
	CU1	0.017	0.873	0.997	0.965	1.031
	CU2	0.019	<0.00	0.914	0.88	0.949
	CU3	0.022	0.181	1.029	0.987	1.074
	CU4	0.014	0.466	0.99	0.963	1.017
	CU5	0.024	0.814	1.006	0.959	1.054
	AV1	0.012	0.052	0.977	0.954	1
	AV2	0.052	0.473	1.038	0.938	1.148
	AV3	0.032	0.602	1.017	0.954	1.084
	AV4	0.019	0.612	0.99	0.954	1.028

	AV5	0.012	0.427	0.99	0.968	1.015
	AV	0.023	0.616	0.988	0.944	1.035
	CV	0.26	0.263	0.971	0.923	1.022
	Amended CU	0.021	0.001	0.933	0.896	0.972
	TS	0.23	>0.000	0.887	0.848	0.928
Top 3 Weighted Categories	CV1	0.008	0.004	0.953	0.937	0.969
	CV2					
	CV3	0.011	0.172	1.015	0.994	1.038
	CV4	0.018	0.689	0.992	0.958	1.128
	CV5	0.017	0.054	1.034	0.999	1.07
	CU1	0.016	0.522	1.01	0.979	1.043
	CU2					
	CU3	0.02	0.66	1.009	0.97	1.074
	CU4	0.022	0.172	1.03	0.987	1.076
	CU5					
	AV1	0.13	0.524	0.992	0.955	1.001
	AV2					
	AV3	0.32	0.564	1.006	0.957	1.084
	AV4	0.021	0.564	0.990	0.953	1.028
	AV5	0.013	0.426	0.990	0.966	1.015
	AV	0.22	0.597	0.908	0.946	1.033
	CV	0.18	0.181	0.976	0.942	1.011
	Amended CU	0.020	0.001	0.934	0.698	0.972
	TS	0.019	>0.000	0.9	0.887	0.937

Additional File Table S13: Cox Regression Model for unweighted, weighted, and weighted top 3 categories, CRC Biomarker scores.						
		SE	Sig.	Exp(B)	95.0% CI for Exp(B)	
					Lower	Upper
Unweighted	AV1	0.005	0.767	1.001	0.992	1.011
	AV2	0.003	0.000	1.021	1.016	1.026
	AV3	0.018	0.453	0.987	0.953	1.022
	AV4	0.010	0.426	1.008	0.989	1.027
	AV5	0.005	0.774	1.001	0.992	1.011
	CV1	0.004	0.002	0.988	0.981	0.996
	CV2	0.004	0.034	1.010	1.001	1.018
	CV3	0.052	0.388	1.046	0.945	1.157
	CV4	0.011	0.247	0.988	0.967	1.009
	CV5	0.007	0.025	0.984	0.971	0.998
	CU1	0.018	0.091	1.030	0.995	1.067
	CU2	0.004	0.001	0.987	0.979	0.995
	CU3	3.814	0.958	0.817	0.000	1440.305
	CU4	0.003	0.000	0.985	0.979	0.991
	CU5	0.008	0.307	0.992	0.976	1.008
	Amended CU	0.005	0.000	0.959	0.949	0.969
	AV	0.011	0.001	1.040	1.017	1.064
	CV	0.009	0.343	0.991	0.974	1.009
	Total Scores	0.010	0.000	0.936	0.918	0.954
Weighted-All	AV1	0.005	0.767	1.001	0.992	1.011
	AV2	0.007	0.000	1.053	1.040	1.067
	AV3	0.035	0.453	0.974	0.909	1.043
	AV4	0.014	0.426	1.011	0.984	1.038
	AV5	0.008	0.774	1.002	0.986	1.019
	CV1	0.005	0.002	0.985	0.976	0.994
	CV2	0.011	0.034	1.024	1.002	1.047
	CV3	0.086	0.388	1.077	0.910	1.276
	CV4	0.013	0.247	0.985	0.959	1.011
	CV5	0.012	0.025	0.974	0.952	0.997
	CU1	0.020	0.091	1.034	0.995	1.074
	CU2	0.010	0.001	0.968	0.949	0.987
	CU3	6.356	0.958	0.714	0.000	########
	CU4	0.008	0.000	0.964	0.949	0.978
	CU5	0.010	0.307	0.990	0.970	1.010
	Amended CU	0.005	0.000	0.958	0.948	0.968
	AV	0.019	0.034	1.040	1.003	1.079

	CV	0.015	0.362	0.986	0.958	1.016
	Total Scores	0.012	0.000	0.913	0.892	0.934
Top 3 Weighted Categories	AV1	0.004	0.752	1.001	0.993	1.010
	AV3	0.035	0.867	1.006	0.939	1.078
	AV4	0.014	0.171	1.020	0.992	1.049
	AV5	0.009	0.353	0.992	0.976	1.009
	CV1	0.004	0.008	0.988	0.980	0.997
	CV3	0.074	0.328	1.075	0.930	1.243
	CV4	0.013	0.414	0.989	0.964	1.015
	CV5	0.011	0.116	0.983	0.962	1.004
	CU1	0.016	0.354	1.015	0.984	1.046
	CU3	5.921	0.956	0.719	0.000	78890.474
	CU5	0.010	0.007	0.975	0.956	0.993
	Amended CU	0.006	0.000	0.956	0.946	0.966
	AV	0.014	0.603	0.993	0.965	1.021
	CV	0.015	0.793	0.996	0.967	1.026
	Total Scores	0.011	0.000	0.910	0.889	0.930

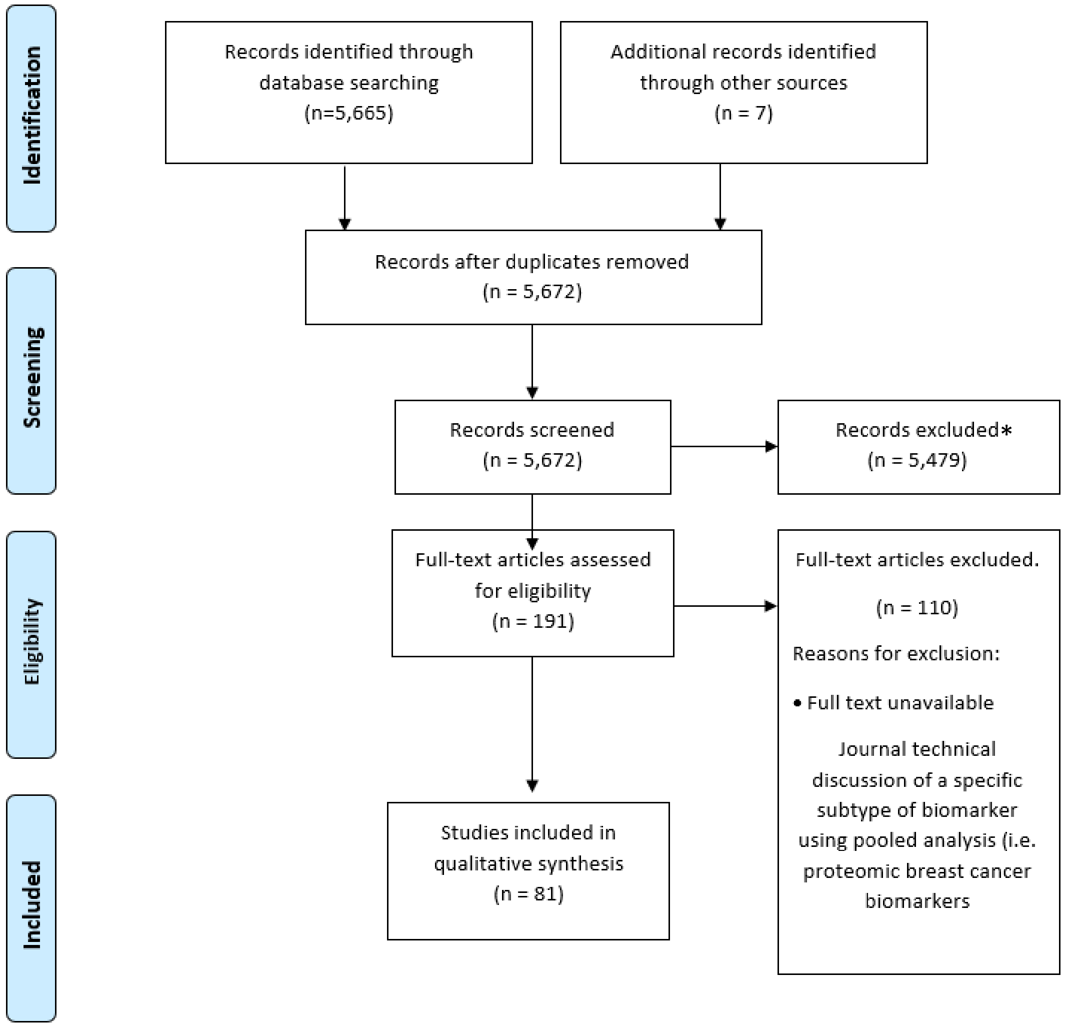

Additional File Figure S1: **PRISMA illustrating study selection for Biomarker criteria checklist**. * Reasons for exclusion include: not written in English Language, conference abstracts, technical biomarker papers, molecular biology primary studies.

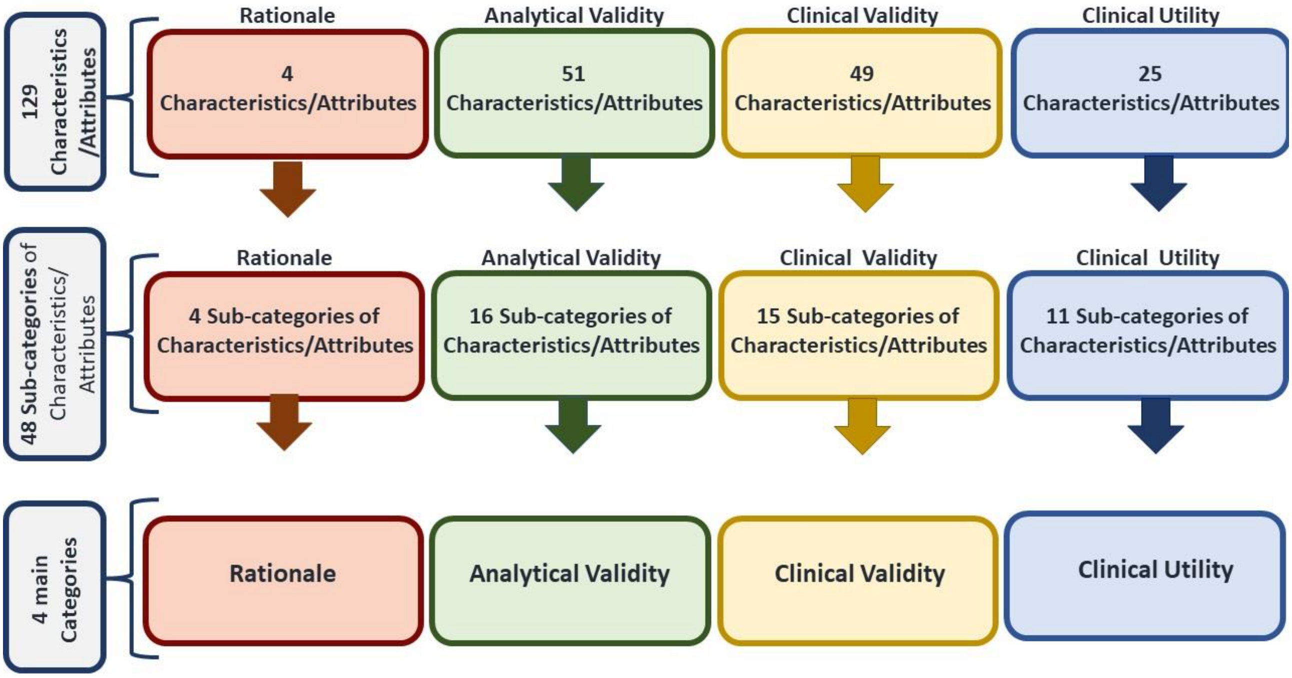

Additional File Figure S2: Categorisation/Grouping of Biomarker toolkit Characteristics. Biomarker characteristics were initially grouped into 48 sub-categories, according to theme, which then merged into four main categories.

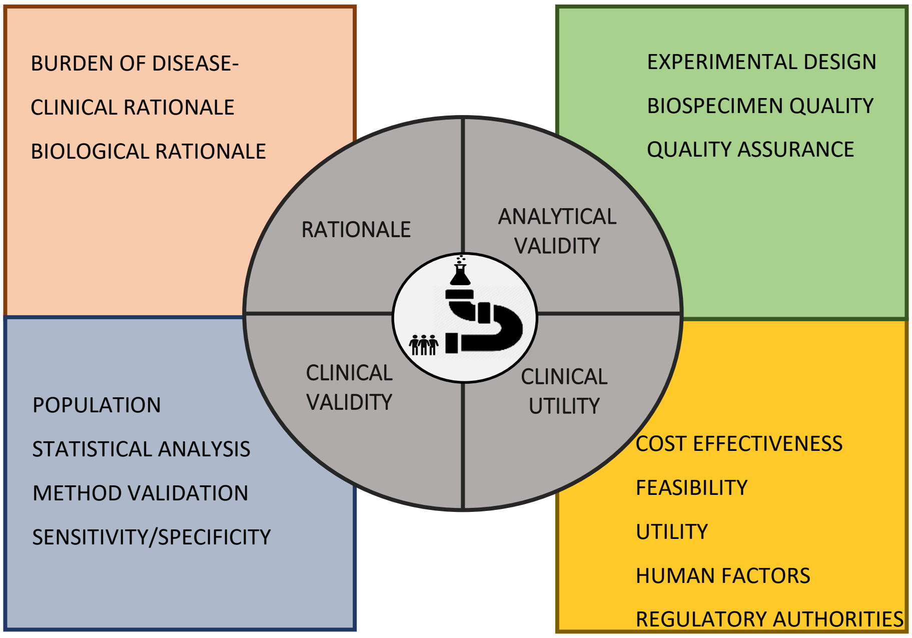

Additional File Figure S2b: Biomarker characteristics associated with successful biomarker clinical implementation These characteristics were identified using a systematic literature search. 125 attributes were identified and separated into four categories: Rationale, Clinical Validity, Clinical Utility and Analytical Validity.

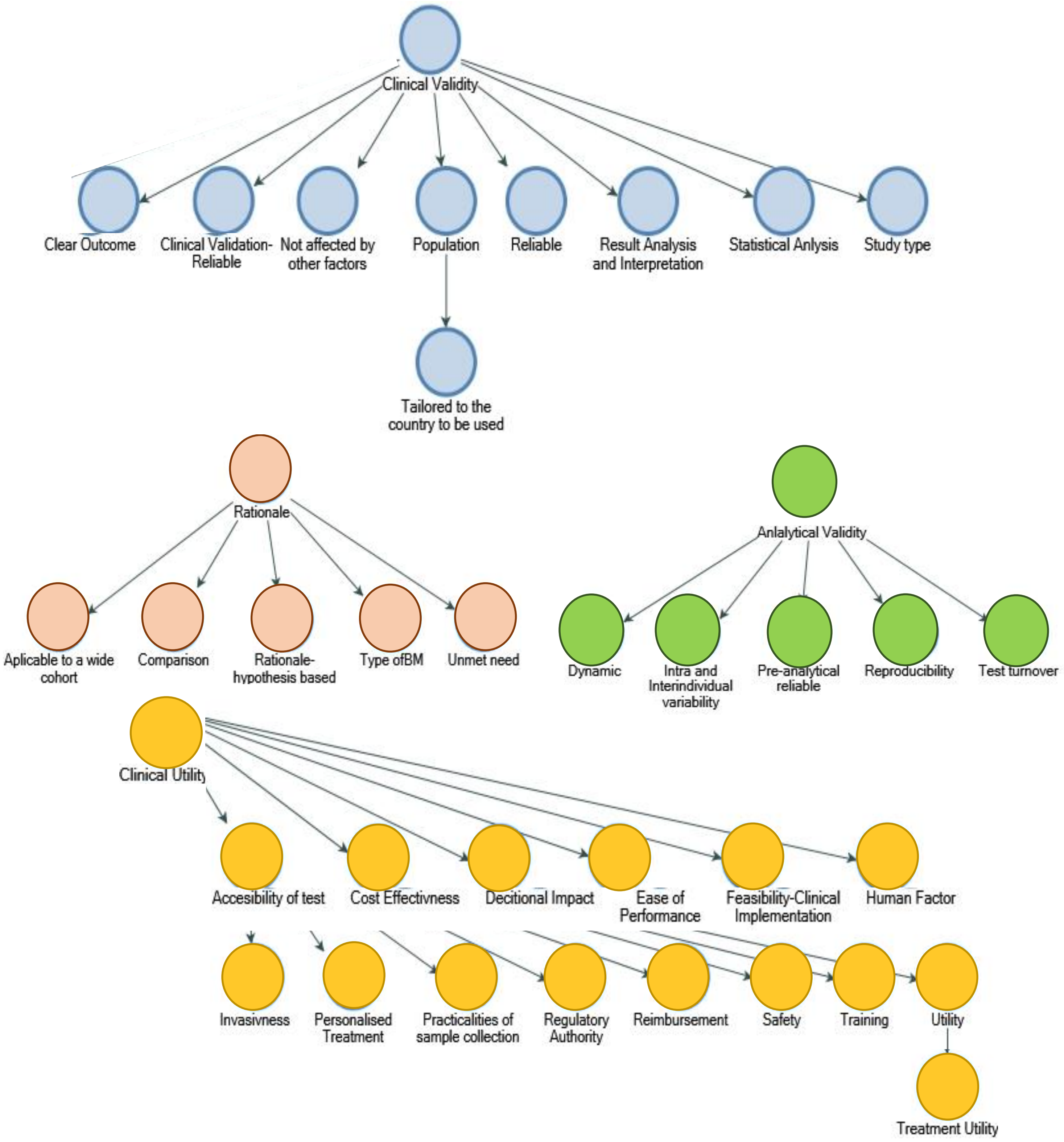

Additional File Figure S3: Themes identified via semi-structured interview thematic analysis. Thematic analysis and figures were constructed using Nvivo12 Pro.

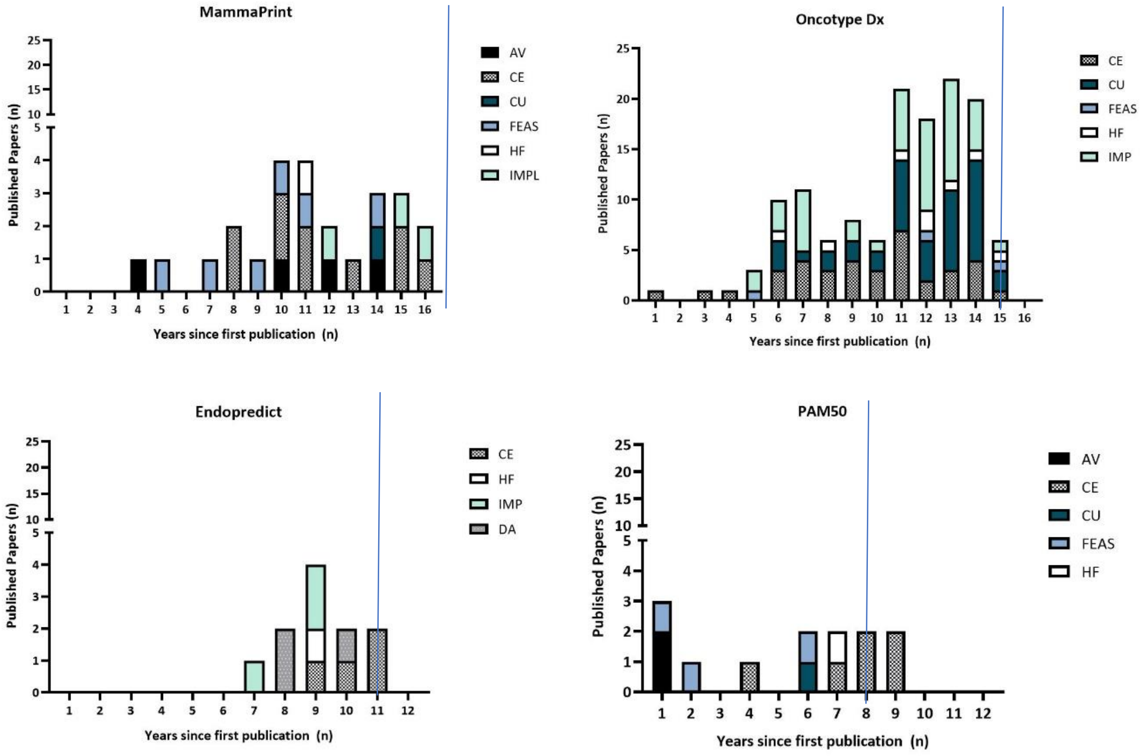

Additional File Figure S4: Successful Biomarker Clinical Utility Studies Stacked Bar chart showing AV, CE, CU, FEAS, HF, IMPL & DA studies in **A)** MammaPrint, **B)** Oncotype Dx, **C)** PAM 50 and **D)** Endopredict

AV: Analytical Validity, **CE**: Cost Effectiveness, **CU**: Clinical Usefulness, **FEAS**: Feasibility; **HF**: Human Factor; **IMPL**: Implementation and **DA**: Decisional Analysis.

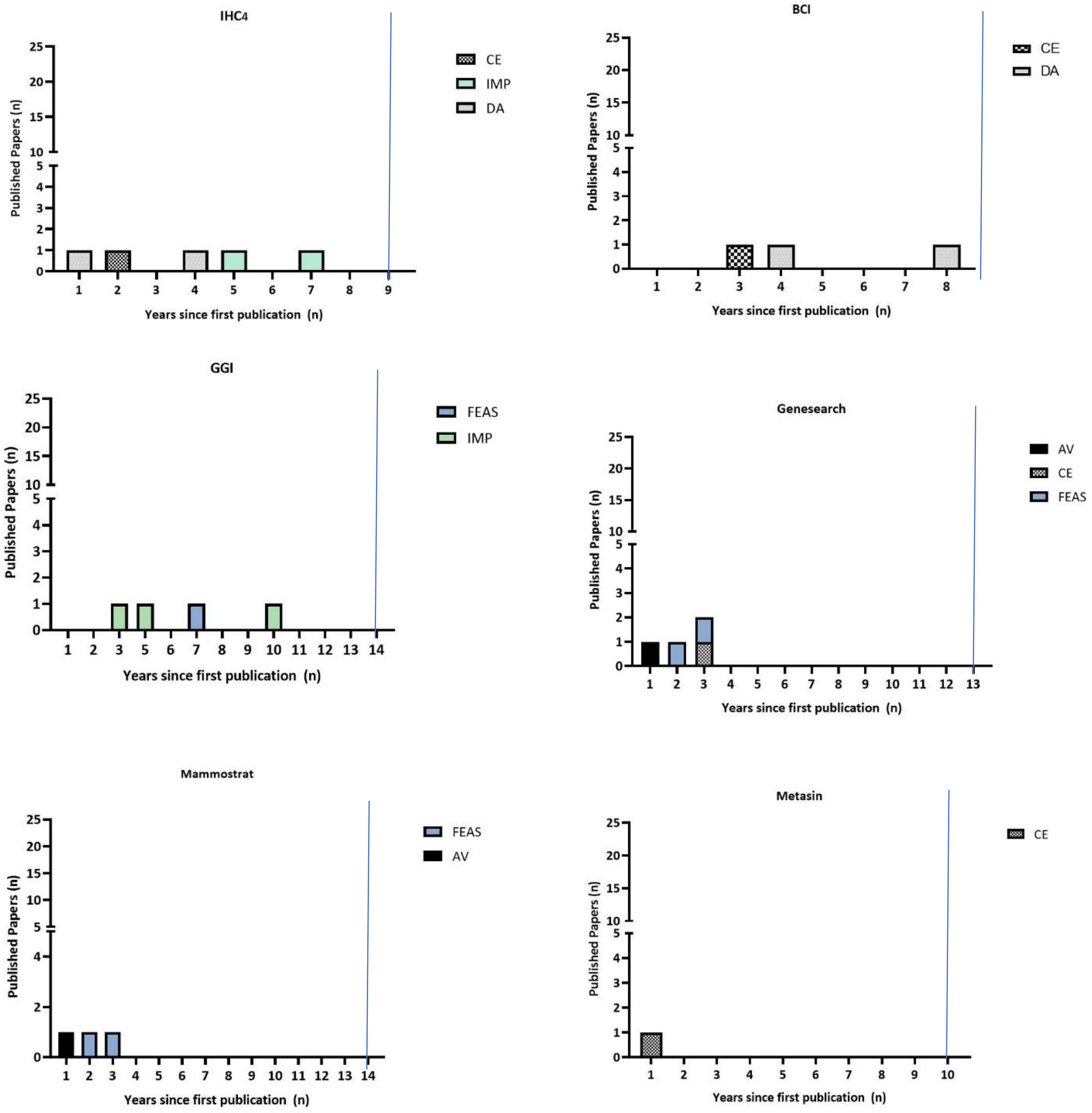

Additional File Figure S5: **Stalled Biomarker Clinical Utility Studies** Stacked Bar chart showing AV, CE, CU, FEAS, HF, IMPL & DA studies in **A)** IHC4, **B)** BCI, **C)** GGI, **D)** GeneSearch, **E)** Mammostrat and **F)** Metasin

AV: Analytical Validity, **CE**: Cost Effectiveness, **CU**: Clinical Usefulness, **FEAS**: Feasibility; **HF**: Human Factor; **IMPL**: Implementation and **DA**: Decisional Analysis.

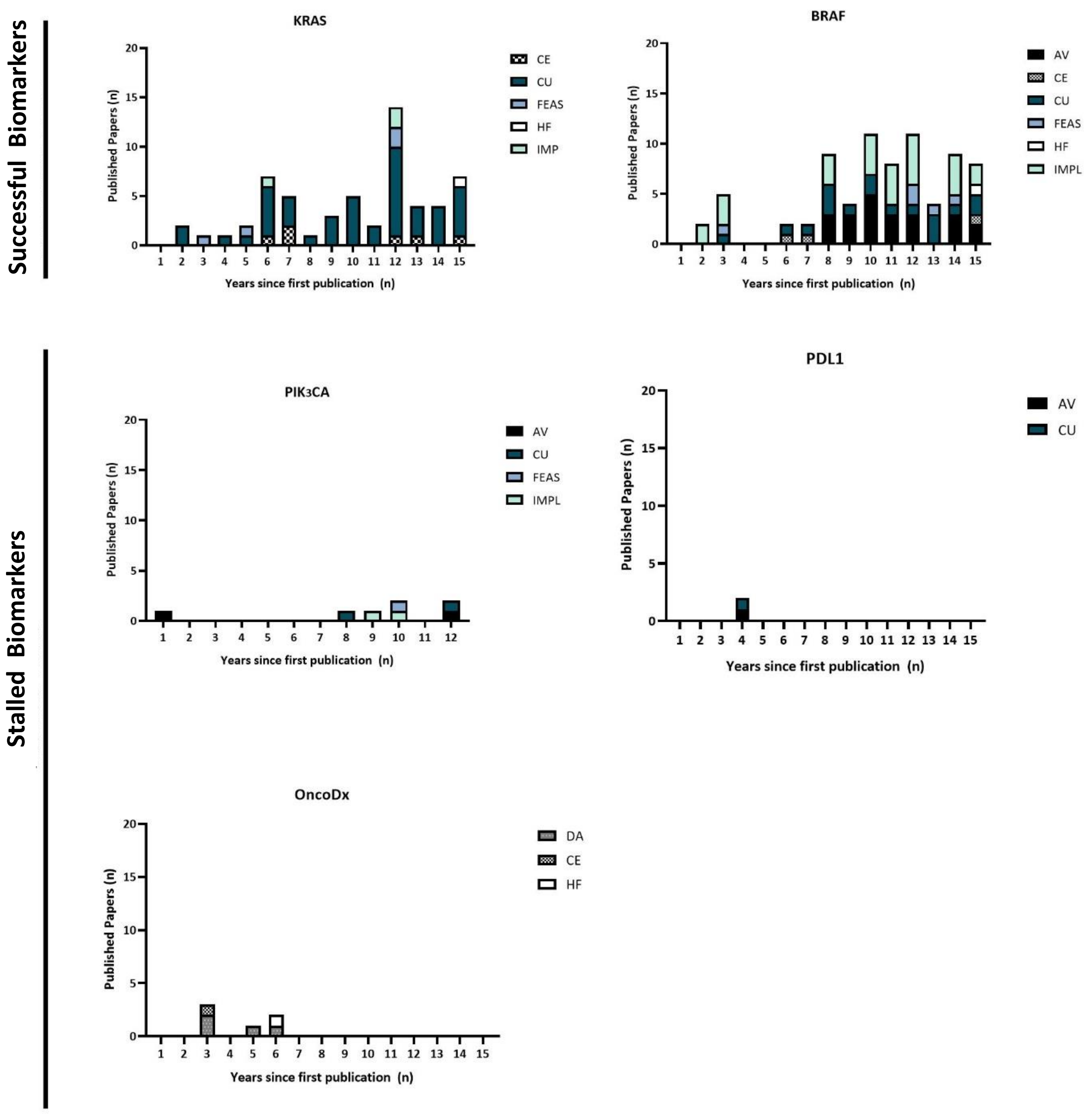

Additional File Figure S6: **Clinical Utility Studies** Stacked Bar showing AV, CE, CU, FEAS, HF, IMPL & DA studies in Staled and Successful Colorectal Cancer Biomarkers.

AV: Analytical Validity, **CE**: Cost Effectiveness, **CU**: Clinical Usefulness, **FEAS**: Feasibility; **HF**: Human Factor; **IMPL**: Implementation and **DA**: Decisional Analysis.

ADDITIONAL FILE: METHODS

A mixed methodology (combination of a qualitative and quantitative approach) was selected to address the main chapter objective and further develop the Biomarker Toolkit checklist. Initially, semi-structured interviews were conducted to allow in-depth exploration and communication of the different themes under the Biomarker checklist, identified by systematic literature search. An online Delphi Survey was also utilised to achieve expert consensus regarding these characteristics while also asking participants to:

i) Prioritise which category of biomarker attributes (Clinical Validity, Clinical Utility, Analytical Validity and Rationale) is more significant at each stage of the biomarker pipeline.

ii) To rank attributes falling under each of these categories, for different biomarker types.

The study methodology was based on grounded theory which was characterised in 1967, by two sociologists, Glasser and Strauss, as the 'theory that was derived from data, systematically gathered and analysed through the research process'. Grounded theory has been described in different ways since its first characterisation, but there are certain core underlying features that remain crucial across all versions including: i) Simultaneous generation and collection of data via surveys, interviews, focus groups and literature, within other sources, ii) Initial coding and category identification, iii) Intermediate coding and subgrouping of codes into core categories and iv) Advance coding, a process in which the researcher interconnects coding between categories in an attempt to build a storyline grounded on the data.

The current study design was developed based on grounded theory with the support of qualitative expert SM.

Semi-Structured Interviews

Participant Recruitment

Participant recruitment for the semi-structured interviews was initiated in September 2019. Participants were purposely recruited based on their expertise in the field of biomarker research. Following up from Huddy et al. (2015), participants were separated in four different groups: clinicians, academic/scientists, industry representatives and cancer patient representatives/carers/survivors. A minimum of 8 participants were interviewed per group as a pragmatic approach, taking into consideration the time scale of this study. Where necessary additional interviews were conducted until thematic saturation was achieved. Participant inclusion criteria involved being older than 18 years old, fitting in one of the previously stated groups and, in the case of academic personnel, having a minimum of three years of experience in the biomarker field. Participant exclusion criteria include vulnerable population e.g., individuals who have a disability/illness that might affect their ability to give consent and non-English speakers. Potential clinicians, scientist and industrial personnel were recruited via e-mail.

Study Protocol for Semi-Structured Interview

Semi-structured interview format enabled a flexible method of data acquisition, through the use of pre-set open-ended questions as an interview guide/basis, allowing the interviewer to adjust the wording of questions, according to participant response. Open-ended questions were introduced to explore beliefs and thoughts of the participants, based on their own experiences. The interview structure and dissemination material were generated by KVS in collaboration with a qualitative research expert (SM), and then verified (MN and CJP). Initially, all participants were introduced to a simplified version of the Biomarker Toolkit checklist shown in **Additional file: Figure S7**. All characteristics detailed in the Biomarker Toolkit checklist were summarised and grouped according to common themes to promote more efficient participant understanding. Interview questions and

dissemination material were adjusted and tailored to be comprehensible to all participant groups including cancer patient representative group, with the support of Imperial PERC.

During the interview the following semi-structured questions were asked:

- What do you think makes a good biomarker? Please list five characteristics linked with a successful biomarker.
- Please have a look in the table overleaf (**Additional file: Figure S7**) which lists biomarker attributes associated with clinical implementation, identified via a systematic literature search. Are there any attributes missing? Why do you think they are important?
- Demographics regarding participant age, sex, occupation, educational level and years of experience were also asked.

Data confidentiality

Data was anonymised, and participants were unidentifiable, as each one was given a unique ID. Interview response were audio recorded and transcribed verbatim, after which the data were immediately encrypted and stored. These data will only be accessible by members of the research team. Electronically transcribed data were subsequently thematically analysed using Nvivo Pro V.10.1.1 software (QSR International, Melbourne, Victoria, Australia). Electronic transcripts were not returned to participants, unless transcript clarifications were needed.

BM ATTTRIBUTE		BM ATRRIBUTE DESCRIPTION
RATIONALE	BURDEN OF DISEASE	Does the BM address a disease of unmet need?
ANALYTICAL VALIDITY	EXPERIMENTAL DESIGN	Are appropriate control groups/reference standards assigned?/ Are the experimental outcomes clearly reported? /Does the design consider blinding to avoid bias?
	BIOSPECIMEN QUALITY	Is the sample appropriately collected, stabilised, stored and transported? / Is the sample collected using a SOP?/ Does the sample utilised address the research question?
	QUALITY ASSURANCE	Are the equipment used appropriately calibrated/assessed for their performance?/ Is there any technical variability?
CLINICAL VALIDITY	SENSITIVITY/SPECIFICITY	Is the BM able to correctly identify target population? e.g. Can the BM distinguish between high and low risk patients with high sensitivity & specificity?
	METHOD VALIDATION	Is the technique used to measure/ assess BM levels validated and standardised? Or is it under development?
	POPULATION	Does the population included in the study address the research question?/ Are exclusion and inclusion criteria clearly stated?/ Will the population selected result in high risk of bias?
	STATISTICAL ANALYSIS	Is the data collected appropriately analysed (Confidence intervals and odds ratio included)?<br>Is the model performance reported (e.g. Survival Analysis)?
CLINICAL UTILITY	HUMAN FACTOR	Is the BM detection method acceptable by the patient and clinician?
	ETHICAL APPROVAL	Is the study ethically approved?
	REGULATORY AUTHORITIES	Is the BM FDA/NICE approved?
	COST EFFECTIVENESS	Does implementation of this BM result in reduced hospital admissions /more expensive tests/more expensive drugs?
	FEASIBILITY	Can it be implemented in clinical practice?
	UTILITY	Can the BM be incorporated in clinical care? Is the technique used to assess BM automated? / Who will benefit from utilisation of this BM?

Additional File Figure S7: Simplified version of the Biomarker Toolkit. This table was shown and discussed with the participants at Q2, during semi-structured interviews.

BM: Biomarker

Data Analysis

Interviews were coded based on predetermined themes, according to the detailed Biomarker Toolkit checklist, while additional emerging themes were added according to participant responses. The interviewer was allowed to contact the participants to clarify sections of the interview, if unclear. Interviews were piloted with four participants (2 clinicians, 2 academics), and 20% of the interviews were coded by a second qualitative researcher (SW). Reporting of semi-structured interviews was conducted following the COREQ checklist.

Delphi Survey Round 1

Participants

Inclusion and exclusion criteria were the same as previously described in the method section. Following up from the semi-structured interview recruitment strategy, a minimum of eight participants were purposely recruited. Potential clinicians, scientists and industrial personnel were purposely recruited via e-mail. An online link of the survey, a digital consent form and participant information leaflet was electronically distributed in a targeted manner with a snowball approach. Reminder emails were sent every two weeks, within the first month, after the initial email invite.

Study Protocol for Delphi Round 1

The online Delphi survey was designed by KVS and reviewed by qualitative expert SM and CJP, using Qualtrics platform. All emergent themes from the systematic literature search and the semi-structured interviews were conveyed in a series of statements in the Qualtrics questionnaire (Qualtrics Labs Inc, Provo, UT). Due to the high number of characteristics in the Biomarker Toolkit checklist (n>120), statements in the checklist were thematically grouped into 51 categories to allow a more efficient review and improve participant usability (Analytical Validity:13, Clinical Validity: 16, Clinical Utility: 17, Rationale: 5).

To address our research questions, the study was separated in three stages:

Stage A: Aimed to reach consensus regarding the characteristics related to successful biomarker translation, using a five point of agreement Likert scale (Disagree, Somewhat disagree, Neutral, Somewhat agree, Agree). Responders were given the chance to add additional characteristics under each subcategory (Clinical Validity, Clinical Utility, Analytical Validity and Rationale), using free text questions at the end of each section.

Stage B: Aimed to prioritise which category of biomarker attributes (Clinical Validity, Clinical Utility, Analytical Validity and Rationale) is more important at each stage of the biomarker pipeline, using a 5 point of importance Likert scale (Not Important (=5), Somewhat Important (=4), Important (=3), Very important (=2), Extremely important (=1)).

Stage C: Aimed to prioritise attributes related to each biomarker type and evaluate whether difference in the type of biomarker results in different attribute prioritisation, using rankings from 1-5, where 1 denotes highest importance while 5 corresponds to the least important.

A maximum of three rounds were allowed, and a consensus threshold was set at 75% agreement amongst participants. Upon round 1 completion, all responses were exported and analysed in Microsoft Excel (2007) while they were graphically presented in GraphPad prism (La JoLa, California, US).

Delphi Round 2

Study Participants

Recruitment approach of Delphi Round 2 was the same as Delphi Round 1.

Study Protocol for Delphi Round 2

Biomarker characteristics in Round 1-Stage A that did not reach consensus during the first phase of the Delphi, were re-assessed during Delphi Round 2. At this stage, potential items were recorded as additional characteristics in the Biomarker checklist, based on participant input in the free text questions of Round 1-Stage A. In this round the results of Delphi-Round 1 -Stage A were shared in an anonymous manner. This allowed participants to reconsider their responses in light of the results, in an attempt to achieve 75% consensus in the remaining items. The Delphi was open from Oct-Dec 2020, and it was expected to take 10 minutes to complete. This round was also kept active for the

extended period of three months, due to the impact of COVID-19 and participant request to extend the deadline.

Ethical Approval

Information provided by the responders was kept anonymised and participant information remained confidential, e.g., name, DOB, etc. Study participation was voluntary, while all potential participants had the right to refuse or withdraw from the study at any given point. In both semi-structured interviews and Delphi, participants were provided a patient information leaflet and were allowed enough time to make an informed decision in respect to their participation in the study (at least two weeks). Both sectors of these studies were approved by the Head of the Department and the Joint Research Compliance.

Score Manual

In Step 1 the average of scores of all attributes addressing Analytical Validity, Clinical Validity & Clinical Utility are generated for each clinical study using the following formulae (equations 1a-c):

Equations 1a-c

$$AV_j = \frac{\sum_{i=1}^{N_1} AV_i^j}{N_1}$$

$$CV_j = \frac{\sum_{i=1}^{N_2} CV_i^j}{N_2}$$

$$CU_j = \frac{\sum_{i=1}^{N_3} CU_i^j}{N_3}$$

j: Study number

J: All studies

i:1-n number of attributes

AV: Analytical validity

CV: Clinical validity

CU: Clinical utility

DA: Decision analysis

N_1: Total number of attributes in the AV category

N_2: Total number of attributes in the CV category

N_3: Total number of attributes in the CU category

AV_i^j : denotes i^{th} attribute of jth study under the AV category

CV_i^j : denotes i^{th} attribute of jth study under the CV category

CU_i^j : denotes i^{th} attribute of jth study under the CU category

IMPL: Implementation Studies

HF: Human Factor

We now illustrate how one uses the formulae in practise. Below you can see a simplified version of the toolkit, with a few of the attributes, as a worked example for score calculation. In the following example, there are 5 studies in total and N_1 is 4, N_2 is 6 and N_3 is 3. As shown in Worked Example Part 1, study 1 is scored based on the reporting of specific attributes. For instance, using "Experimental design" as an example: if experimental design is clearly reported in the journal, then the study scores "1", otherwise "0" is assigned.

At the first step, the average of the scores from all attributes, per study, per category is calculated. This is repeated for all clinical studies regarding each biomarker being assessed.

Worked Example Part 1:

BM ATTRBUTE		Study 1	Study 2	Study 3	Study 4	Study x		
DISEASE BURDEN	RATIONALE							
ANALYTICAL VALIDITY	EXPERIMENTAL DESIGN	1	1					
	BIOSPECIMEN QUALITY	0	1					
	QUALITY ASSURANCE	1	1					
	CELL CULTURE	1	1					
SUM		3	4	4	2	1		
AVERAGE		3/4	4/4	4/4	2/4	1/4	0.70	Step 3
						Step 1		
CLINICAL VALIDITY	ADVERSE EVENTS	1	1					
	POPULATION DETAILS	1	0					
	MISSING DATA	0	0					
	RANDOMISATION	0	0					
	SENSITIVITY/SPECIFICITY	1	1					
	STATISTICAL MODELLING	1	1					
SUM		4	3	5	2	4		
AVERAGE		4/6	3/6	5/6	2/6	4/6	0.60	
						Step 1		
CLINICAL UTILITY	COST EFFECTIVENESS	0	0					
	FEASIBILITY	1	0					
	UTILITY	1	0					
SUM		2	0	0	1	2		Step 2
AVERAGE		2/3	0/3	0/3	1/3	2/3	0.33	
						Step 1		

Worked Example Part 1: Step 1 of score calculations using Equations 1a-c. The sum of scores per study is calculated and then divided by the number of attributes in that category. i.e. Sum of scores for study 1, Analytical Validity related attributes is “3” and the total number of attributes is “4”. Thus, as seen in the orange row the average for this sector is: “3/4”. The same is repeated for each study in each main category of attributes

Attributes included in Clinical Utility section including Cost Effectiveness, Feasibility and Impact of biomarker application were not addressed by clinical studies. Therefore, Clinical Utility score generated from clinical studies was adjusted, taking into consideration their publication date, based on the presence of Implementation, Feasibility, Cost-effectiveness, Utility and Human Factor studies (equation 2 d). Score '100' was assigned to primary studies addressing biomarker Implementation/Feasibility/Cost-effectiveness/Utility/Human factors; otherwise '0' score was assigned. Thus, in step 2 the Non-Adjusted Clinical Utility score is amended using equation 2. Below the equation you can see the calculation for the worked example. Taking into consideration that:

i) study 1 is published in 2008

ii) the biomarker studied in the worked example has a Cost effectiveness study (2006), a decisional analysis study (2003) and a human factor study (2009) associated with it.

As seen in equation 2, the non-adjusted % Clinical Utility score from step 1 is used together with positive score for the Cost Effectiveness and Decisional Analysis study that was published before 2008 (100% was assigned, as cost effectiveness and decisional analysis studies were present- see equation 2 worked example). The Human Factor study was issued after study 1 was published; thus it was not used to amend the Clinical Utility score of Study 1.

Equation 2:

$$Adj.CU_j = \frac{(CU_j * 100) + U_j + CE_j + IMP/FEAS_j + DA_j + HF_j}{6}$$

where the present quantities are defined as follows:

$$U_j = \begin{cases} 100, & if\ present \\ 0, & otherwise \end{cases}$$

$$CE_j = \begin{cases} 100, & if\ present \\ 0, & otherwise \end{cases}$$

$$IMP/FEAS_j = \begin{cases} 100, & if\ present \\ 0, & otherwise \end{cases}$$

$$DA_j = \begin{cases} 100, & if\ present \\ 0, & otherwise \end{cases}$$

$$HF_j = \begin{cases} 100, & if\ present \\ 0, & otherwise \end{cases}$$

j: Study number

J: All studies

i:1-n number of attributes

AV: Analytical Validity

CV: Clinical Validity

CU: Clinical Utility

DA: Decision analysis

N_1: Total number of attributes in the AV category

N_2: Total number of attributes in the CV category

N_3: Total number of attributes in the CU category

AV_i^j : denotes i^{th} attribute of jth study under the AV category

CV_i^j : denotes i^{th} attribute of jth study under the CV category

CU_i^j : denotes i^{th} attribute of jth study under the CU category

IMPL: Implementation Studies

HF: Human Factor

Worked Example Part 2:

BM ATTRBUTE		Study 1	Study 2	Study 3	Study 4	Study x	
DISEASE BURDEN	RATIONALE						
ANALYTICAL VALIDITY	EXPERIMENTAL DESIGN	1	1				
	BIOSPECIMEN QUALITY	0	1				
	QUALITY ASSURANCE	1	1				
	CELL CULTURE	1	1				
SUM		3	4	4	2	1	
AVERAGE		3/4	4/4	4/4	2/4	1/4	0.70
CLINICAL VALIDITY	ADVERSE EVENTS	1	1				
	POPULATION DETAILS	1	0				
	MISSING DATA	0	0				
	RANDOMISATION	0	0				
	SENSITIVITY/SPECIFICITY	1	1				
	STATISTICAL MODELLING	1	1				
SUM		4	3	5	2	4	
AVERAGE		4/6	3/6	5/6	2/6	4/6	0.60
CLINICAL UTILITY	COST EFFECTIVENESS	0	0				
	FEASIBILITY	1	0				
	UTILITY	1	0				
SUM		2	0	0	1	2	
AVERAGE		2/3	0/3	0/3	1/3	2/3	0.27

Step 3

Step 1

Step 2

$$Adjusted\ CU\ score = AVERAGE\left(\left(\left(\frac{2}{3}\right) * 100 \right) + 100 + 100 + 0 + 0 + 0 \right)$$

Worked Example Part 2: This uses equation 2 to adjust the Clinical Utility score based on the presence of a cost effectiveness study (2006) and a decisional analysis study (2003). "2/3" represents the score of Clinical Utility, for study 1 (=2) (worked example 1), divided by the total number of attributes (=3). 2/3 is then multiplied by 100 to become a percentage. "100" is assigned for the presence of i) cost effectiveness study and a ii) decisional analysis study. "0" is assigned for utility, feasibility/implementation, and human factor studies as there were none conducted prior to study 1 publication date (2008).

In step 3, the sum of all of the attributes, for all of the studies identified in the biomarker of interest is calculated using the formulae:

Equations 3a-c:

$$AV\ score = \frac{\sum_{j=1}^{J}(AV_j)}{J} \qquad CV\ score = \frac{\sum_{j=1}^{J}(CV_j)}{J} \qquad Adjusted\ CU\ Score = \frac{\sum_{j=1}^{J}(Adj.CU_j)}{J}$$

Worked Example Part 3:

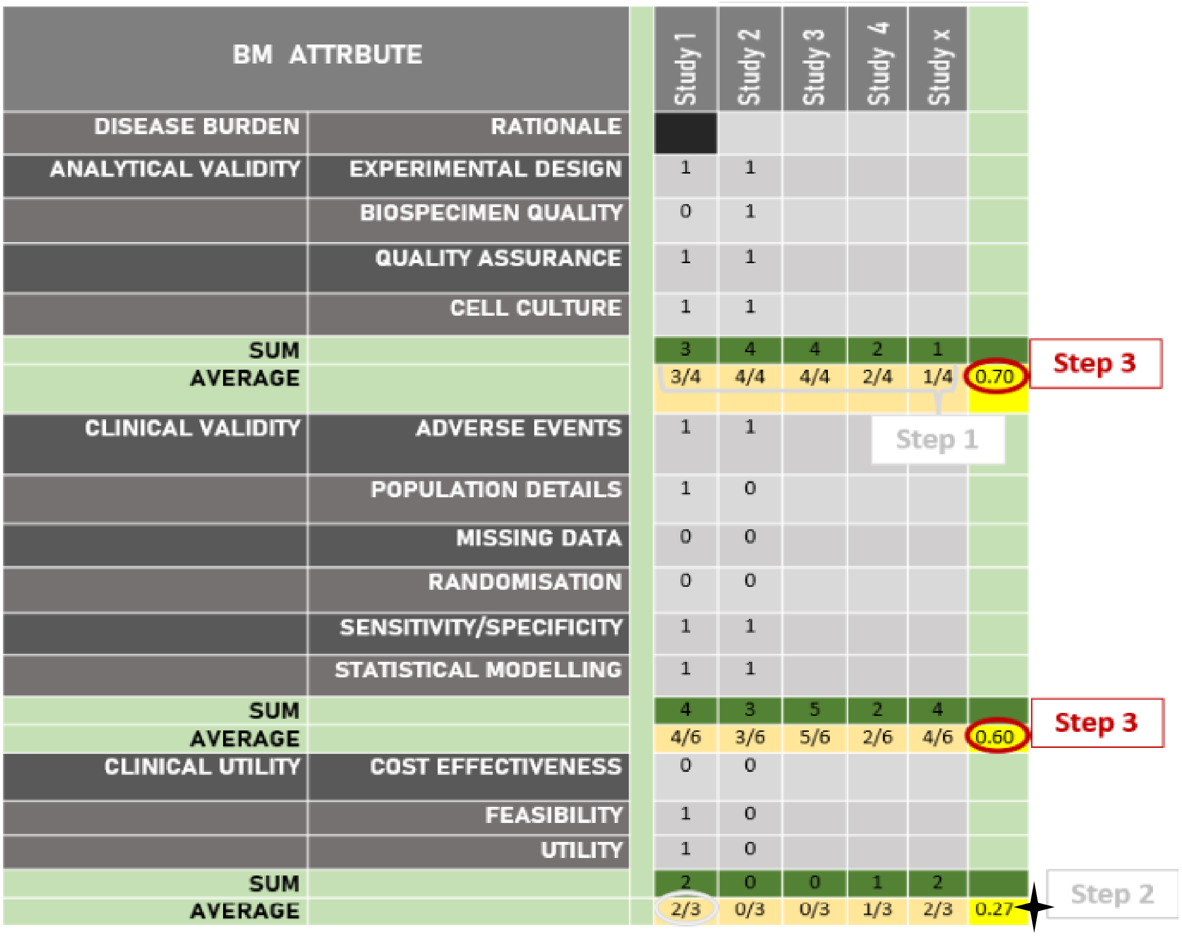

BM ATTRBUTE		Study 1	Study 2	Study 3	Study 4	Study x	
DISEASE BURDEN	RATIONALE						
ANALYTICAL VALIDITY	EXPERIMENTAL DESIGN	1	1				
	BIOSPECIMEN QUALITY	0	1				
	QUALITY ASSURANCE	1	1				
	CELL CULTURE	1	1				
SUM		3	4	4	2	1	
AVERAGE		3/4	4/4	4/4	2/4	1/4	0.70
CLINICAL VALIDITY	ADVERSE EVENTS	1	1				
	POPULATION DETAILS	1	0				
	MISSING DATA	0	0				
	RANDOMISATION	0	0				
	SENSITIVITY/SPECIFICITY	1	1				
	STATISTICAL MODELLING	1	1				
SUM		4	3	5	2	4	
AVERAGE		4/6	3/6	5/6	2/6	4/6	0.60
CLINICAL UTILITY	COST EFFECTIVENESS	0	0				
	FEASIBILITY	1	0				
	UTILITY	1	0				
SUM		2	0	0	1	2	
AVERAGE		2/3	0/3	0/3	1/3	2/3	0.27

Worked Example Part 3: Stage 3 of score calculations using *Equations 3 (a-d).* ✦ *Star indicates that raw Clinical Utility scores, that are used to generate the adjusted Clinical Utility score using equation 2.*

In step 4, the overall score is calculated (equation 3) by averaging the scores identified in step 3 assuming that variables are of equal importance.

Equation 4: $Assumption: Variables\ are\ of\ equal\ importance$

$$Overall\ score = \frac{CV\ Score(\%) + AV\ Score(\%) + Adjusted\ CU\ score(\%)}{3}$$

For instance, in the example above we have:

$$Overall\ Score\ = \frac{(0.70 * 100) + (0.60 * 100) + (0.45 * 100)}{3}$$

Worked Example Part 4: Equation 4 was used to calculate the overall score from the worked example. These three % scores are divided by three to achieve an average between the three categories which corresponds to the overall score.

It should be noted that if the Biomarker test in the selected publications was conducted under commercial laboratories, the scores under the relevant subcategory (Analytical Validity) were adjusted based on relevant publications content, where applicable. For example if an assay optimisation publication was identified for biomarker X, then “1” would be assigned in the attribute “Did the study report study optimisation?”, in every publication that used this specific optimised assay.

Statistical Analysis Justification

Cox regression (or proportional hazards regression) is used to formulate predictive model for time-to-event data. For the purpose of this analysis "event" was considered to be biomarker stalling. In this paper Cox-Regression was used as it enables the evaluation of the effects of several variables, taking into consideration the effect of time. In this case study publication date was considered in the model, in addition to other variables including: i.e., Clinical Validity, Clinical Utility and Analytical Validity scores in addition to biomarker type. Therefore, the influence of variables on time-to-event occurrence could be investigated.

A logistic regression was performed to assess the relation of each biomarker's: **i)** sub-category score, **ii)** Analytical Validity score, **iii)** Clinical Validity score, **iv)** Clinical Utility score and **v)** Total % score with Biomarker implementation status. Since implementation status is a binary measure, logistic regression was used, which also allows the assessment of how well the set of variables can predict the categorical dependant variable (biomarker success) and provide a summary of accuracy % regarding the classification of your cases. This can be used to determine the % of correct predictions generated by the model.
